# Initial specialist validation of clinical decision support recommendations from a machine learning-enabled digital cognitive assessment

**DOI:** 10.3389/fneur.2026.1806000

**Published:** 2026-06-17

**Authors:** Ali Jannati, Claudio Toro-Serey, Marissa Ciesla, Emma Chen, David Bates, John Showalter, Sean Tobyne, Alvaro Pascual-Leone

**Affiliations:** 1 Linus Health, Inc., Boston, MA, United States; 2 Department of Neurology, Harvard Medical School, Boston, MA, United States; 3 Hinda and Arthur Marcus Institute for Aging Research and Deanna and Sidney Wolk Center for Memory Health, Hebrew SeniorLife, Boston, MA, United States

**Keywords:** Alzheimer’s disease, clinical decision support, core cognitive evaluation, dementia, digital clock and recall, digital cognitive assessment, machine learning, mild cognitive impairment

## Abstract

**Introduction:**

Disease-modifying therapies for Alzheimer’s disease (AD) heighten demand for scalable tools enabling primary care providers (PCPs) to detect cognitive impairment and triage patients for appropriate evaluation. The brief tablet-based Linus Health Core Cognitive Evaluation (CCE) integrates the Digital Clock and Recall (DCR) and Life and Health Questionnaire (LHQ) to generate clinical decision support (CDS) and decision-tree pathways.

**Methods:**

We conducted a retrospective specialist content-validity study (June 15–27, 2023) using a modified RAND/UCLA Appropriateness Method. Five board-certified cognitive/behavioral neurologists independently rated CDS recommendations and nine predefined pathway parts for patients aged ≥55 across 21 de-identified reports. Items scored 1–9 were summarized as pooled medians with interquartile ranges; medians ≥7 indicated appropriateness. Agreement among experts was quantified using ICC [2,k] and ICC [2,1].

**Results:**

All cognitive-impairment recommendations met the threshold. All seven borderline/impaired-DCR pathways were appropriate (median 7–8). Two pathways fell below threshold: cognitively unimpaired individuals with Green DCR scores (median 6) and a preliminary anti-amyloid treatment referral pathway (median 5). Agreement was moderate per patient [median ICC(2,k) = 0.61] and lower for individual diagnostic-concern recommendations [median ICC(2,k) = 0.25], reflecting specialist heterogeneity on borderline non-cognitive items and ceiling effects on high-rated items.

**Conclusion:**

Cognitive neurologists judged CCE-derived CDS appropriate for PCP workup and referral decisions in older adults with suspected cognitive impairment. Findings support initial content validity of assessment-linked CDS, identify refinement priorities in low-risk and emerging-therapy pathways, and motivate planned PCP appropriateness and prospective implementation studies.

## Introduction

Dementia is a major and growing public health challenge, with an estimated 55 million people worldwide living with Alzheimer’s disease (AD) or related dementias in 2020—a number projected to reach 78 million by 2030 as populations age (1, 2). Early detection of cognitive impairment and dementia is increasingly critical, particularly in light of recent breakthroughs in disease-modifying therapies (DMTs) for AD (3–6). After nearly two decades without new approved treatments, the U.S. Food and Drug Administration (FDA) recently approved the monoclonal antibodies lecanemab and donanemab to target amyloid pathology and slow disease progression (7, 8). Both have demonstrated a modest ability to slow cognitive and functional decline in patients with mild cognitive impairment (MCI) or early dementia due to AD (3, 4), including in real-world applications (9, 10). Accordingly, identifying individuals in the earliest stages of cognitive decline has become increasingly important so that eligible patients can benefit from emerging interventions before substantial neurodegenerative change accumulates. Early diagnosis also allows patients and families to plan for the future and initiate treatments and risk-reduction strategies that may help maintain function and potentially slow further decline (1, 11, 12).

Despite the importance of early detection, cognitive impairment often goes unrecognized in primary care. Primary care providers (PCPs) are usually the first point of contact for older adults, yet most PCPs do not perform routine cognitive screening during visits (13, 14). Surveys indicate that while PCPs generally agree on the importance of screening for MCI and dementia, they report conducting formal cognitive assessments in less than half of patients over 60 years old (14). This gap is attributable to multiple well-documented barriers—time constraints during brief primary care visits, competing health priorities, and lack of training or confidence in administering cognitive tests (14–16). Until recently, a pervasive sense that little could be done for early cognitive impairment further reduced the incentive for aggressive case-finding (17, 18). Consequently, more than 90% of individuals with MCI remain undiagnosed (19), and diagnoses are often delayed until dementia has progressed to more advanced stages, at which point functional impairment is pronounced, therapeutic options are few, and caregiver burden is high (20–22).

Digital cognitive assessments (DCAs) have emerged as a promising solution to facilitate the timely detection of MCI and dementia in primary care (14, 16, 23–29). DCAs leverage tablets, voice and speech features, stylus input, and machine learning (ML) algorithms to streamline cognitive testing and automate result interpretation (30–36), reducing administrative time and the need for specialized neuropsychological training and thereby making cognitive screening more feasible in busy clinics. One such tool is Linus Health’s Core Cognitive Evaluation (CCE™), a brief (7–8-min) tablet-based DCA developed to address barriers to cognitive screening in primary care (28). The CCE comprises the Digital Clock and Recall (DCR™; 2.5–3 min) (32, 33, 36), the Life and Health Questionnaire (LHQ™; 4–5 min), and clinical decision support (CDS) functionality that provides actionable next steps tailored to the individual patient. An emerging literature additionally highlights visuospatial memory and spatial navigation as among the earliest-affected cognitive functions in preclinical and prodromal AD (37); these domains are not the principal target of the present CCE configuration but are relevant to next-generation digital cognitive assessment, as discussed below.

While the introduction of tools such as the CCE is promising, it raises an important question: are the CDS recommendations generated by this system clinically appropriate? Given that these recommendations will be used by non-specialist providers to make real-world patient care decisions, it is crucial to verify that the advice aligns with what an expert would recommend in those scenarios. Before novel clinical decision support algorithms are widely adopted, their content validity and safety must be established (38–41). We therefore sought to validate the clinical appropriateness of the CCE’s automated recommendations for cognitive impairment by having experienced cognitive and behavioral neurologists systematically review and rate the CCE’s guidance—its recommended evaluations, referrals, and diagnostic workups—for appropriateness in primary care. This expert-review step constitutes the first of a planned multi-stage validation program; the design of the next-stage primary-care provider validation is described in the Discussion.

## Methods

### Study design and objective

We conducted a retrospective expert review (June 15–27, 2023) to evaluate the clinical appropriateness of recommendations and decision pathways generated by the CDS functionality embedded in the Linus Health Core Cognitive Evaluation (CCE) for patients aged 55 years and older. The methodological approach was aligned with established expert-panel consensus processes to streamline the translation of evidence into practice and with commonly applied appropriateness-rating conventions in expert-panel studies (42, 43). Specifically, we used a modified RAND/UCLA Appropriateness Method (RAM) (44). The primary objective was to quantify (i) the median expert rating for each CDS recommendation (organized by diagnostic concern) and (ii) the median expert rating for each predefined CDS pathway branch, and to assess inter-rater agreement using intraclass correlation coefficients (ICCs) (45).

### Core cognitive evaluation (CCE)

The Linus Health CCE is a brief digital cognitive evaluation comprising (i) the Digital Clock and Recall (DCR) and (ii) the Life and Health Questionnaire (LHQ) (13, 35). The DCR is an FDA-listed Class II medical device and a digital, process-based evolution of the Mini-Cog (32–34, 36). The Mini-Cog is a brief (~3-min) paper-based cognitive screen that combines a three-word delayed-recall task with a clock-drawing test; it is widely used in primary care because it is rapid, free, and validated for the detection of dementia, although its sensitivity for mild cognitive impairment and its inter-rater reliability for clock scoring are limited (46, 47). The DCR was designed to address these limitations by introducing standardized digital administration and ML-enabled scoring (32–34, 36). The roughly three-minute assessment is completed on an iPad with an Apple Pencil and can be administered by medical assistants without specialized training.

Administration proceeds in three steps:

- *Immediate recall*: Three unrelated words are read aloud by the iPad, which the participant repeats to confirm encoding. Responses are recorded without time limits and primarily support attention/hearing checks and set up the later delayed recall task.
- *Digital clock drawing test (DCTclock™)*: The participant completes two stylus-based clock drawings—first drawing a clock from memory set to 11:10 (command) and then copying an on-screen clock showing the same time (copy). Beyond the final drawings, the software captures completion time and detailed process-level metrics, assessing visuospatial and visuoconstructional skills, executive planning, information processing speed, and motor control.
- *Delayed recall*: The participant then recalls the original three words. Spoken responses are recorded, and the application captures both accuracy and speech/acoustic features to assess episodic memory, which is often affected early in Alzheimer’s disease.

The DCR passively captures high-resolution drawing and speech data to generate quantitative process features. During clock drawing, it records time-stamped stylus trajectories to derive kinematic and timing measures (e.g., speed, stroke metrics, total duration, on-tablet time, pauses, and latencies) as well as spatial organization from the final drawing (e.g., symmetry/circularity, numeral and hand placement, and screen positioning). It also analyzes spoken language during recall for timing and acoustic markers (e.g., response latency, pauses, pitch, jitter, shimmer, and speech rate). Together, these inputs yield roughly ~2000 features used by ML models beyond traditional scoring (36).

The DCR scoring is fully automated. Delayed-recall audio is transcribed using an automatic speech recognition (ASR) pipeline tuned for short, single-word utterances; recognized tokens are then matched against the three target words by a rules-based scorer (0–3). Immediate-recall audio is recorded but not scored and is available for clinician review. Clock drawings are scored by a hybrid ML and rules-based engine that parses time-stamped pen strokes into clock elements (face, numerals, hands, corrections/extraneous marks) and computes a clock score on a 0–100 metric (31, 48) that is mapped to the Mini-Cog-aligned 0–2 component score. Both the captured recall audio and the transcription, together with the rendered command and copy clock images and component-level subscores, are presented in the clinician-facing report so that the clinician can verify or override the automated score. The performance of the DCR scoring across a range of speaker characteristics, including age, sex, and self-reported race/ethnicity, has been examined in prior work (32, 49, 50), including among stroke patients (51). In contrast, performance in speakers with strong non-U.S.-English accents and in patients with speech-motor disorders such as dysarthria or post-stroke aphasia has not yet been systematically evaluated and requires further validation. The ~2000 process features extracted by the DCR are consumed by the scoring ML models to produce the calibrated DCR tier (Green/Yellow/Red) but are not directly surfaced to the clinician; instead, the report exposes interpretable, clinician-facing elements (clock images, subscores, audio playback, recall accuracy, and a plain-language summary of diagnostic concerns), and the downstream CDS recommendations are produced by a rule-based engine that consumes these interpretable elements rather than the raw feature vector.

The total DCR score ranges from 0–5, combining Delayed Recall (0–3) and the clock score (0–2), aligned with the Mini-Cog but with a finer underlying clock metric (0–100) (31). Results are reported in three tiers: Green (4–5) indicates no significant impairment; Yellow (2–3) suggests borderline findings warranting monitoring or intervention; and Red (0–1) indicates a high likelihood of impairment consistent with MCI or dementia (32, 33). The report includes the tier/score plus component-level details (and playback) to support rapid interpretation. An ML model using multimodal DCR features has been validated for accurate identification of cognitive impairment (36). A summary of DCR feature families with example features is provided in Supplementary Table S1.

The LHQ is a brief questionnaire designed to identify modifiable lifestyle and health risk factors associated with cognitive decline and dementia. It was adapted from the findings of the Lancet Commission (52) and the Barcelona Brain Health Initiative (BBHI) multidomain survey (49, 50, 53, 54) and includes domains relevant to dementia risk and prevention (e.g., mood, physical activity, cardiovascular factors). The LHQ also supports a dementia risk score derived from a proprietary algorithm that integrates multiple risk and protective factors informed by validated risk constructs such as Lifestyle for Brain Health (LIBRA) and Cardiovascular Risk Factors, Aging, and Incidence of Dementia (CAIDE) (55–61). Specifically, the dementia risk score integrates seven risk and protective factors: physical inactivity, depression, cognitive activity, cardiovascular risk burden (incorporating hypertension, hyperlipidemia, diabetes, smoking, and obesity), sleep quality, social engagement, and alcohol intake. Item-level weights are derived from a data-driven calibration against the LIBRA (56) and CAIDE (59) frameworks, and the resulting score is dichotomized at a prespecified threshold to trigger the LHQ “high-risk” CDS recommendation. A summary of LHQ inputs and the weighting overview is provided in Supplementary Table S2.

### Clinical decision support (CDS): recommendations and pathways evaluated

**Figure 1:**
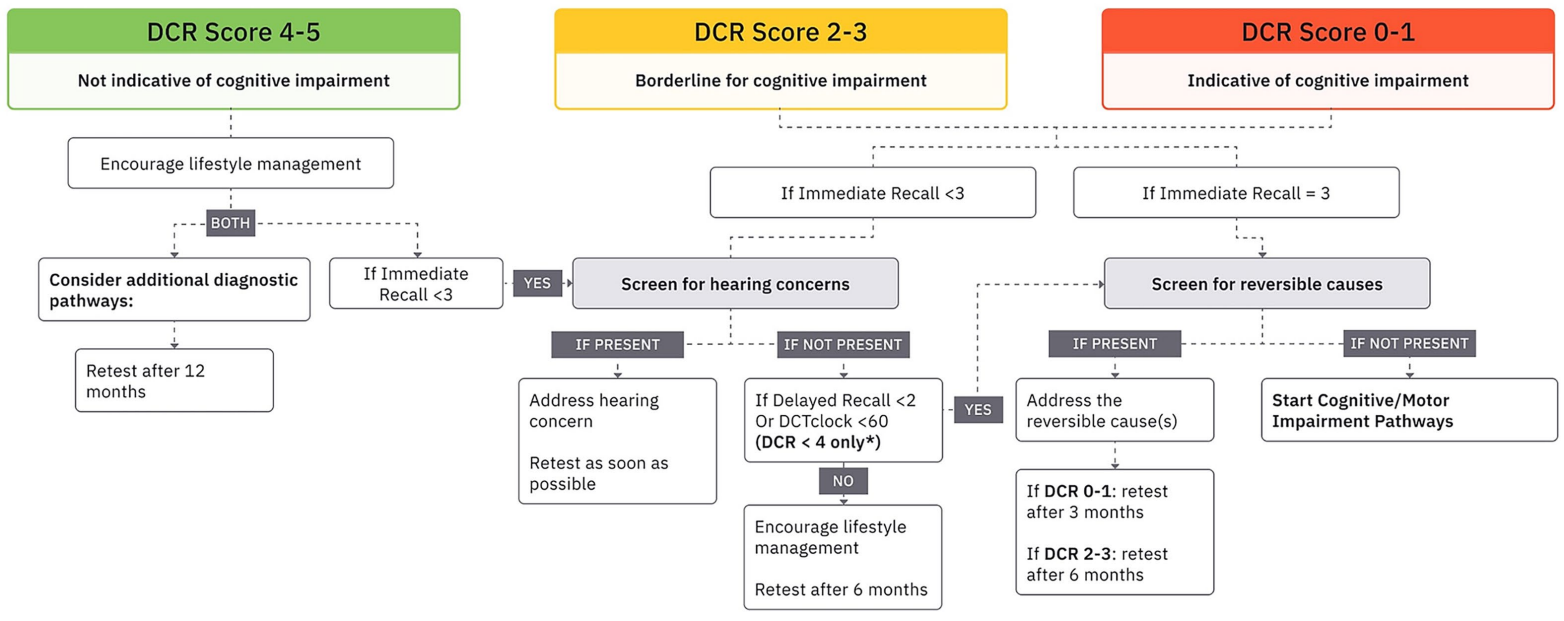
Clinical decision support (CDS) decision-tree pathways were evaluated by the expert panel. Cognitive-impairment pathways, conditional on Digital Clock and Recall (DCR) performance. Recommendations are stratified by DCR total score category (Green: not indicative of cognitive impairment; Yellow: borderline; Red: indicative of cognitive impairment) and refined using additional conditional nodes, including immediate recall performance, screening for hearing concerns, and evaluation for reversible causes. Pathway outputs include lifestyle guidance, retesting intervals (3, 6, or 12 months depending on risk strata), and initiation of diagnostic pathways for cognitive or motor impairment when indicated.

The CDS functionality of the CCE generates actionable, patient-specific recommendations conditional on DCR performance and LHQ responses, using rule criteria grounded in the presence or absence of cognitive impairment, as determined by specific DCTclock subscales and verbal memory performance, to inform the likely cognitive phenotype. CDS outputs include (i) recommendation sets linked to diagnostic concern flags (e.g., executive/vascular, verbal memory, mixed-domain, executive/mixed, verbal memory/mixed impairment, as well as Parkinsonism, tremor, and cholinergic pathway concerns) with associated diagnostic and referral suggestions, and (ii) structured decision-tree pathways corresponding to DCR category strata (Green, Yellow, Red) and additional conditional nodes (e.g., immediate recall strata and reversible-cause screening) (Figure 1); a distinct pathway addressing referral considerations for anti-amyloid evaluation (e.g., lecanemab, donanemab), including blood biomarker testing where appropriate, was also included for expert review to reflect evolving care pathways for MCI or early dementia due to AD (5, 6, 62).

### Anti-amyloid pathway

**Figure 2:**
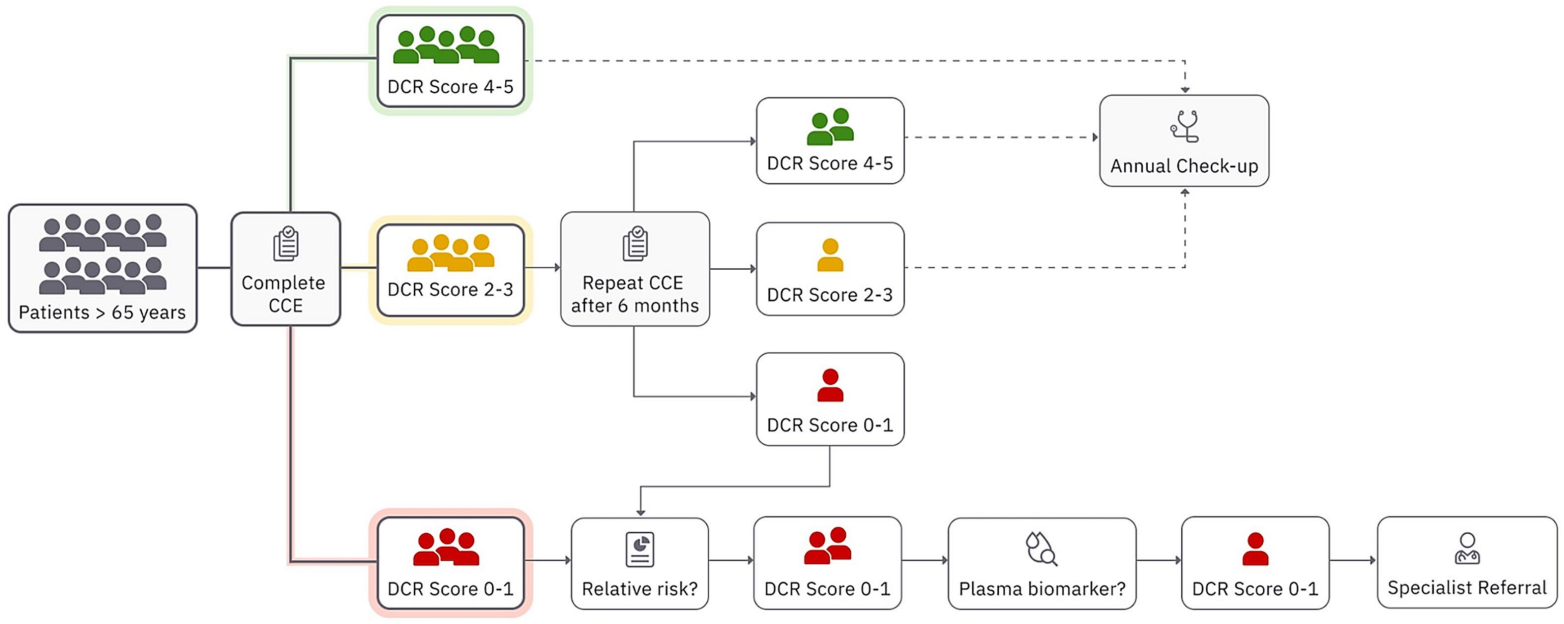
Anti-amyloid clinical decision-support pathway, as presented to the expert panel (June 2023). Flow diagram illustrating the recommended care pathway for patients aged ≥ 65 years following completion of the Linus Health Core Cognitive Evaluation (CCE). After CCE administration, patients are stratified into one of three Digital Clock and Recall (DCR) tiers indicated by icon color: green (DCR total score 4–5; cognition within normal range; top track), yellow (DCR score 2–3; borderline; middle track), and red (DCR score 0–1; suggestive of amnestic mild cognitive impairment [MCI] or early dementia of the Alzheimer type; bottom track). Green-tier patients are routed to routine annual follow-up (dashed arrow to annual check-up). Yellow-tier patients undergo a CCE re-test at 6 months and are re-stratified into the same three tiers: those who remain green or yellow are returned to annual follow-up, whereas those who decline to red are escalated to the anti-amyloid pathway. Red-tier patients—and yellow-tier patients who decline at re-test—enter the anti-amyloid pathway (bottom row), in which the following decision nodes are evaluated in sequence: (i) Confirmation that the cognitive impairment is consistent with MCI or early dementia and that no obvious exclusion criterion is documented in the Life and Health Questionnaire (LHQ; “Relative risk?”); (ii) screening for major contraindications, including anticoagulation, recent stroke, prior macrohaemorrhage, uncontrolled hypertension, and APOE ε4 homozygosity flagged as elevated risk of amyloid-related imaging abnormalities (ARIA); (iii) blood-based biomarker testing—e.g., plasma p-tau217 and/or the Aβ42/40 ratio (“Plasma biomarker?”)—and/or referral to a cognitive specialist for confirmatory cerebrospinal fluid or amyloid-positron-emission-tomography testing; and (iv) where biomarkers are consistent with Alzheimer pathology, referral (“Specialist Referral”) for eligibility evaluation for anti-amyloid therapy (e.g., lecanemab), including APOE genotyping and a baseline magnetic resonance imaging scan for ARIA screening. Solid arrows indicate the recommended forward path; dashed arrows indicate routine annual follow-up. Note. The pathway is depicted as it was presented to the expert panel in June 2023 and therefore predates the published Appropriate Use Recommendations (AUR) for lecanemab (5) and donanemab (6); an updated, post-AUR version of the pathway, with explicit blood-biomarker triage, eligibility filtering for APOE ε4 homozygotes, and MRI exclusion criteria, is provided as Supplementary Figure S1.

Given the clinical relevance of this pathway, the anti-amyloid pathway is described here in detail and depicted in Figure 2. The pathway, as it was presented to the panel in June 2023, was triggered when a patient had a DCR score in the Yellow or Red range that was suggestive of amnestic MCI or early dementia of the Alzheimer type, and no obvious exclusion criterion was documented in the LHQ. Decision nodes were: (i) confirm cognitive impairment consistent with MCI/early dementia; (ii) screen for major contraindications (e.g., anticoagulation, recent stroke, history of macrohaemorrhage, uncontrolled hypertension, or APOE ε4 homozygosity flagged as elevated ARIA risk); (iii) order blood-based biomarker testing (e.g., plasma p-tau217 or the Aβ42/40 ratio) and/or refer to a cognitive specialist for confirmatory CSF or amyloid-PET testing; (iv) if biomarkers are consistent with Alzheimer pathology, refer for eligibility evaluation for lecanemab, including APOE genotyping and a baseline MRI for ARIA screening. Caveats were that the pathway was preliminary at the time of review and predated the published Appropriate Use Recommendations (AUR) for lecanemab (5) and donanemab (6). The updated pathway, as implemented post-AUR, is depicted in Supplementary Figure S1, which summarizes the changes (explicit blood-biomarker triage, refined APOE-based eligibility, MRI exclusion criteria, and ARIA monitoring schedule).

### Selection of patient reports and pathway materials

Patient-facing CCE outputs were sampled from 912 completed reports generated in routine clinical settings between August 1, 2022, and December 1, 2022, and available within the Linus Health clinical platform. Sampling was randomized but weighted to match the expected frequency distribution of CDS recommendations, ensuring that each CDS recommendation type appeared at least three times in the expert-review set. Using this sampling approach, 21 de-identified CCE reports were selected for review. This approach ensured each CDS recommendation was rated at least 15 times in total, providing sufficient representation across recommendation categories to support systematic evaluation. All experts reviewed the same set of 21 reports.

In addition to patient-level reports, experts reviewed nine predefined CDS pathway components representing key decision-tree branches of the “Linus Clinical Pathways” framework (as shown in Figure 1). These pathway parts included: (1) the overall pathway; (2) Green pathway; (3) Yellow pathway; (4) Yellow pathway with immediate recall <3 and hearing concerns including the reversible-causes node; (5) Yellow pathway with immediate recall = 3 and downstream nodes; (6) Red pathway; (7) Red pathway with immediate recall <3 and hearing concerns, including the reversible-causes node; (8) Red pathway with immediate recall = 3 and downstream nodes after reversible-cause screening; and (9) the anti-amyloid pathway. Expert ratings across these nine pathway parts ensured a systematic evaluation of the entire decision tree, including both primary categorical branches (Green, Yellow, and Red) and critical decision points within each branch.

### Clinical expert panel: eligibility, recruitment, and participation

A Clinical Expert Panel (CEP) was assembled to provide independent specialist review. Inclusion criteria required: (i) board certification in neurology in the U.S. with specialty practice in cognitive/behavioral neurology; (ii) affiliation with an academic institution; (iii) active evidence-based clinical practice focused on cognitive disorders; and (iv) no current or prior affiliation, collaboration, or financial interest in Linus Health to mitigate potential conflicts of interest. Additional desired qualifications included contributions to clinical guidelines, authorship of clinical textbooks on neurocognitive disorders, research experience, and leadership roles in relevant professional societies.

A panel size of five to nine raters is consistent with RAND/UCLA Appropriateness Method conventions (44), which support robust median ratings and acceptable reliability in this range when panelists are homogeneous in subject-matter expertise. We deliberately prioritized depth of relevant expertise—board certification in cognitive/behavioral neurology, academic affiliation, and the strict no-conflict criterion above—over a larger but less specialized panel; this eligibility profile was a material constraint on the size of the eligible pool.

Seven neurologists agreed to serve as experts in the study; two withdrew due to competing obligations. Five experts completed ratings within the study timeline and constituted the analytic panel for this report. Experts were compensated via honoraria for their time. Experts were instructed to evaluate the risks and benefits of each recommendation and pathway in accordance with accepted best clinical practices and current specialty knowledge. Review time for each item, as well as the total review time, was left to the experts’ discretion. Panel characteristics are summarized in aggregate in Supplementary Table S3; the panel included experts practicing at academic medical centers across multiple U.S. states, with a range of post-fellowship experience and, in a subset of cases, clinical activity in safety-net or underserved settings.

### Ethical oversight, privacy, and data governance

All study procedures were reviewed and approved as exempt by Advarra Center for IRB Intelligence. The investigation was conducted in accordance with ethical principles outlined in the Declaration of Helsinki. The study used only de-identified, redacted patient reports generated previously in routine clinical care; therefore, no *de novo* informed consent was needed for this retrospective expert-review study. Clinical sites contributing data had provided prior authorization to Linus Health to use de-identified patient data for product improvement and evaluation. All source data and expert ratings were stored in secure, access-controlled cloud storage.

Patient reports were de-identified using a standardized redaction procedure (Adobe Acrobat Pro) that removed patient names, month/day of birth, and provider identifiers while retaining birth year (to preserve patient age). No protected health information (PHI) or personally identifiable information (PII) was included in the materials distributed to experts.

### Expert review materials and procedures

Each expert received a standardized review packet comprising:

1. A PDF for each de-identified patient report, including: DCTclock metrics (total and subscores), immediate and delayed recall scores, an image of the final clock drawing output, and the CDS recommendations and associated decision-tree nodes.
2. A companion PDF listing diagnostic indications and their associated CDS recommendations to support consistent interpretation across experts.
3. A CDS pathway PDF depicting the nine pathway parts to be rated.

### Data capture, quality assurance, and completeness checks

To minimize data-entry burden and reduce transcription errors, experts recorded ratings directly in the provided PDFs using embedded drop-down fields. After the experts returned their completed PDFs, the study staff conducted a completeness review and then entered the ratings into a master spreadsheet for analysis. A second study staff member independently verified all entries against the source PDFs. If any rating fields were missing, study staff contacted the expert to obtain complete ratings. All included experts provided complete ratings for all 21 reports and all nine pathway parts by the end of data collection. The completed PDFs, the rating data collected from the experts, and the master spreadsheet containing the ratings and comments from all experts were stored in Linus Health’s secure cloud storage system behind the company firewall.

### Outcomes and statistical analysis

#### Primary outcomes

Primary outcomes were:

1. The panel-pooled median appropriateness rating for each CDS recommendation category (i.e., by diagnostic concern flag), computed as the median of the full pool of individual expert ratings across all 5 raters × all triggering reports for that category.
2. The panel-pooled median appropriateness rating for each CDS pathway part (i.e., node) was computed as the median of the pool of individual expert ratings across the 5 raters for that pathway.

Following standard RAND/UCLA Appropriateness Method practice (44), pooled medians of 7–9 were prespecified as the “clinically appropriate” range, 4–6 as equivocal/uncertain, and 1–3 as inappropriate for clinical use. The interquartile range (IQR) was reported as the companion dispersion measure for each pooled median. Per-rater medians and IQRs are reported in Supplementary Table S4.

#### Inter-rater agreement

Inter-rater agreement was quantified using the intraclass correlation coefficient (ICC) as described by Shrout and Fleiss (45). We calculated both ICC (2, k) and ICC (2,1) under a two-way random-effects model for absolute agreement: the former describes the reliability of the mean/median panel judgment, and the latter that of a single-rater judgment. ICC values with 95% bootstrap confidence intervals were computed for (i) each diagnostic concern category, and (ii) per-patient bundles. Interpretation was anchored to the Koo and Li (63) bands: <0.5 poor, 0.5–0.75 moderate, 0.75–0.9 good, >0.9 excellent. When ratings were invariant across experts for an item (e.g., all experts provided the same score), ICC values were undefined, and the descriptive distribution was reported instead. Complete ICC tables with 95% CIs for every diagnostic concern and every per-patient bundle are provided in Supplementary Tables S9, S10.

#### Descriptive analyses and exploratory views

To characterize the distribution of ratings and identify systematic effects, we additionally summarized ratings (i) per expert rater, (ii) per patient report, and (iii) per diagnostic concern and pathway. Given the expert panel size and the descriptive validation objective, no hypothesis testing was prespecified; analyses focused on medians, dispersion, and agreement indices, consistent with expert-panel validation studies (42, 44).

#### Qualitative analysis of low-rated items

To understand drivers of low ratings, the free-text rationale comments that experts entered alongside their ratings were extracted and thematically coded. Themes were summarized at the diagnostic-concern and pathway-part levels.

## Results

### Clinical expert panel characteristics

Five clinical experts participated in the appropriateness review. All were board-certified cognitive/behavioral neurologists affiliated with academic institutions and recognized for expertise in cognitive neurology. Each expert had an active evidence-based clinical practice and no conflicts of interest related to the digital assessment tool. The panelists independently evaluated the risks and benefits of each CDS recommendation based on commonly accepted best clinical practices, using a standardized 9-point rating scale (1 = not appropriate to 9 = extremely appropriate). Aggregate panel characteristics, including U.S. state, institution type, post-fellowship experience, and safety-net/underserved-practice activity, are summarized in Supplementary Table S3.

### CDS recommendation ratings

**Figure 3:**
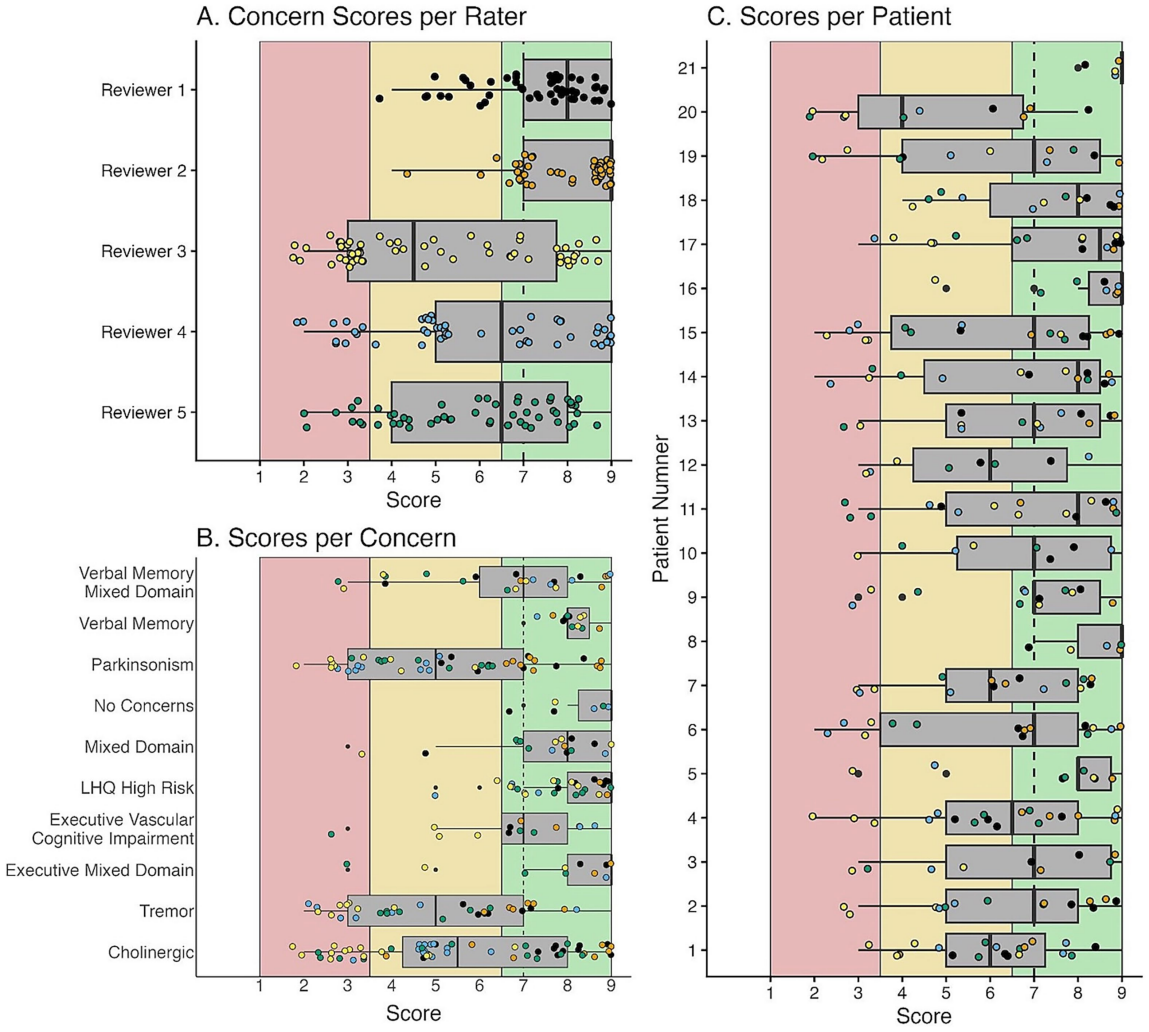
Expert appropriateness ratings of clinical decision support (CDS) recommendations generated for diagnostic concerns identified by the CCE. Five neurologists rated each CDS recommendation on a 1–9 appropriateness scale (overall panel-pooled median, 7; IQR 5–9); the background shading denotes median ratings of 1–3 (red, clinically inappropriate), 4–6 (yellow, equivocal/uncertain), and 7–9 (green, clinically appropriate), and the vertical dashed line denotes the appropriateness-level cutoff. Each boxplot is overlaid with a per-rater color-coded jittered scatter plot (Rater 1–5) and a median bar. **(A)** Distribution of scores by rater. Only Reviewer 3 had a median below 5 (4.5). Median and IQR can be found in Supplementary Table S4. **(B)** Distribution of scores by diagnostic concern category, showing consistently high ratings (median ≥7) for all concerns related to cognitive impairment, lower mid-range ratings (median ~5–5.5) for Parkinsonism, Tremor, and Cholinergic Pathway Impairment, and uniformly high ratings (all ratings 8–9) for the Life and Health Questionnaire (LHQ) high-risk profile. Summary statistics are available in Supplementary Table S5. **(C)** Distribution of ratings by patient. Boxplots show median and interquartile range; whiskers indicate range with outliers plotted as points. Median bars and translucent IQR bands are shown over the boxplots; all individual ratings are plotted as per-rater-colored points. Each patient presented a different combination of concerns, and most were deemed appropriate on average. Summary statistics are available in Supplementary Table S6.

The overall panel-pooled median across all individual recommendation ratings was 7 (IQR 5–9), meeting the threshold for clinical appropriateness (42, 44). Figure 3A shows the distribution of scores per expert: four out of five neurologists had a median recommendation rating at or above 5, and only Reviewer 3’s median fell slightly below 5 (median = 4.5, IQR = 3–7.75). This indicates that, on average, raters scored the recommendations in the upper appropriate range. Specific summary statistics are provided in Supplementary Table S4.

In terms of ratings by diagnostic concern category (Figure 3B), the panel-pooled median across all concern types was 7 (IQR 5–9; all summary statistics are available in Supplementary Table S5). Per-concern ICC values and 95% CIs are reported in Supplementary Table S9. In general, CDS recommendations were rated highly across most diagnostic concerns, with median scores of 7 or higher for all cognitive impairment-related categories (e.g., executive function/vascular impairment, memory impairment, mixed-domain cognitive deficits). In contrast, three recommendation categories stood out for receiving lower ratings: those addressing Parkinsonism, Tremor, and Cholinergic Pathway Impairment, each with median appropriateness scores in the mid-range (approximately 5.0–5.5), falling below the “appropriate” threshold. Notably, the recommendation regarding a high dementia risk profile based on the LHQ received uniformly high ratings from all experts (individual scores of 8 or 9), reflecting strong consensus on its clinical appropriateness.

Because each patient report generated a unique subset of recommendations (depending on which concerns were flagged), we also examined the panel’s ratings aggregated by patient case. As shown in Figure 3C, the panel-pooled median rating per patient was 7 (IQR 5–9; summary statistics are provided in Supplementary Table S6). Thus, on a per-patient basis, the recommended actions were generally deemed clinically appropriate. The lowest median patient-level rating was observed for a case (Patient 20) that triggered only the Tremor and Parkinsonism concerns, consistent with the lower scores given to those specific recommendations. All other patient cases had median recommendation ratings of 7 or higher, indicating that the expert panel considered the combination of recommendations provided for most patients clinically appropriate.

### CDS pathway ratings

The experts also evaluated the appropriateness of the higher-level CDS pathways — the decision tree branches that group recommendations based on the patient’s cognitive assessment results. The panel-pooled median rating across all pathways was 7 (IQR 6–7) out of 9. Summary statistics for ratings by reviewer and by pathway are provided in Supplementary Tables S7, S8, respectively.

All seven pathways related to cognitive impairment (i.e., those triggered by abnormal DCR scores in either the Yellow or Red range) received panel-pooled medians of 7 or higher (median = 7, IQR 6–7), meeting the criterion for clinical appropriateness. In contrast, two other pathways fell below the appropriateness threshold: (1) the pathway for cognitively unimpaired individuals (the “Green” DCR score pathway) had a panel-pooled median of 6 (IQR 6–7); (2) the preliminary anti-amyloid (lecanemab) eligibility pathway, depicted in Figure 2 and described in Methods, (which identifies patients for potential anti-amyloid therapy workup) received a panel-pooled median of 5 (IQR 5–7). These two pathways were the only ones rated as less appropriate by the panel, likely reflecting greater uncertainty about their practical benefit and, for the anti-amyloid pathway, its preliminary status at the time of expert review (June 2023), which predated the published Appropriate Use Recommendations (5, 6).

**Figure 4:**
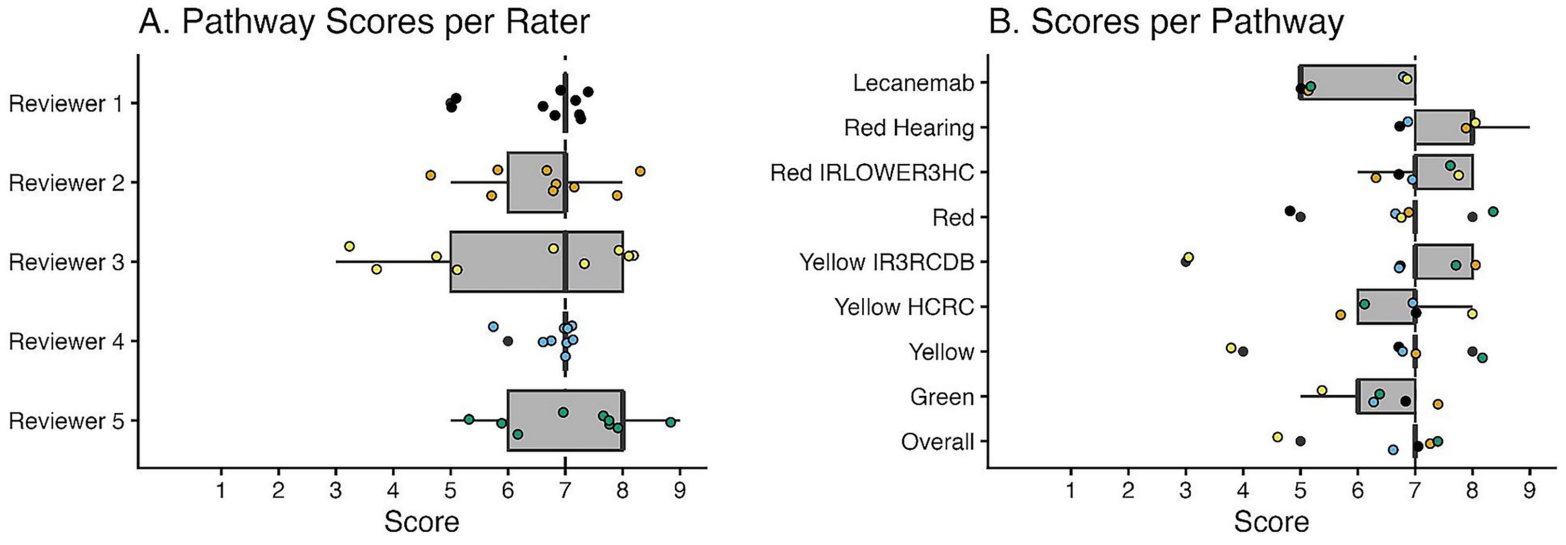
Pathway ratings per rater **(A)** and per pathway **(B)**. Boxplots are overlaid with individual ratings colored by the reviewer who submitted them. Dashed lines show the appropriateness threshold (median ≥7). On average, all raters agreed that pathways are clinically appropriate. The exceptions, shown on the right plot, were pathways for unimpaired individuals (Green) and a preliminary anti-amyloid (lecanemab) pathway. On the y-axis, pathway labels are abbreviated as follows: Red IR3DB (Immediate recall of 3 with downstream branches) refers to the pathway triggered by a red DCR score (0–1) and an Immediate Recall score equal to 3; Red IRLOWER3HC (Immediate recall lower than 3, with hearing concern) refers to the pathway triggered by a red DCR score and an Immediate Recall score lower than 3 (with hearing concerns and reversible-causes nodes); Yellow IR3RCDB (Immediate recall 3, reversible causes and downstream branches) refers to the pathway triggered by a yellow DCR score (2–3) and an Immediate Recall score equal to 3 (with reversible-causes and downstream branches); Yellow HCRC (Hearing concerns and reversible with reversible causes) refers to the pathway triggered by a yellow DCR score with an Immediate Recall score less than 3 (with hearing-concerns and reversible-causes nodes).

Figure 4A illustrates the pathway ratings per rater: each of the five experts had an average (median) pathway score of at least 7, indicating that, individually, all raters generally found the set of pathways appropriate overall. Figure 4B shows the median score for each pathway, highlighting lower agreement in the lower panel for the Green (unimpaired) and anti-amyloid (lecanemab) pathways than for the cognitive impairment–focused pathways, which consistently receive high ratings.

### Inter-rater reliability

Across the individual diagnostic-concern recommendations, agreement between raters varied considerably. The median ICC(2,k) for ratings of individual concerns was 0.25 (IQR 0.05–0.49; 95% CIs reported per category in Supplementary Table S9), indicating only poor-to-low concordance [Koo and Li bands (63)] on average across recommendation categories. When the three lowest-rated categories (Tremor, Parkinsonism, and Cholinergic Pathway Impairment) were excluded, the median ICC across the remaining concerns increased to 0.33, suggesting that these lower-rated items exhibited the greatest variability in expert opinions. One recommendation—the LHQ high dementia risk recommendation—received identical or near-identical scores from all five experts (all ratings were 8 or 9), which resulted in essentially zero variance and an undefined ICC for that item.

Examining reliability at the level of complete patient cases revealed greater consensus among the expert panel members. ICC(2,k) values calculated for each patient’s set of recommendations were substantially greater than those for individual concerns (see Supplementary Table S10 for the summary statistics of ICC values per patient). The median ICC (2,k) across patients was 0.61 (IQR 0.42–0.75), within the “moderate” band (0.50–0.75); the corresponding ICC(2,1) was 0.24 (IQR 0.13–0.37). In other words, the neurologists were more consistent in their appropriateness ratings when considering all recommendations together in the context of a patient’s presentation, even though their agreement on any single specific recommendation varied more widely. This pattern indicates that, taken as a whole for a given patient, the CCE-generated CDS recommendations achieved moderate consensus among the expert panel, with greater per-item variability for borderline or non-cognitive recommendations. For pathways, the ICC across raters was 0.031 (95% CI = −0.12, 0.41) for ICC (2,1) and 0.13 (95% CI = −0.12, 0.77) for ICC (2,k).

### Qualitative themes from low-rated items

Thematic analysis of expert free-text rationale identified three principal themes underlying ratings ≤5. First, some raters expressed concern that clock-drawing-derived flags for Parkinsonism or tremor were insufficient on their own to justify a movement-disorders workup without a brief in-clinic motor examination. Second, raters reported that the rationale for the cholinergic-pathway concern was insufficiently transparent, with several requesting clearer explanation of how component-placement patterns on the DCTclock map to suspected basal forebrain cholinergic involvement. Third, ratings of the anti-amyloid pathway clustered around its preliminary status relative to the Appropriate Use Recommendations (AUR) for lecanemab (5) and donanemab (6) published after the review window. These themes are taken up in the Discussion and have informed concrete refinements to the CDS, including the addition of an intermediate motor-screen step, clearer text and rationale for the cholinergic concern, and replacement of the preliminary anti-amyloid pathway with the AUR-aligned version shown in Supplementary Figure S1.

## Discussion

All CDS recommendations related to cognitive impairment were rated as clinically appropriate by our expert panel, with median ratings of 7 or higher on a 1–9 scale. This indicates specialist endorsement that the CCE-based suggestions for evaluating and managing possible cognitive disorders (e.g., assessing reversible causes, pursuing further cognitive testing, ordering relevant labs or imaging, and referring to specialists) are clinically appropriate. In contrast, a few recommendations outside the core cognitive domain received more lukewarm ratings. In particular, the CDS concerns for tremor, Parkinsonism, and cholinergic pathway impairment had median scores around 5–5.5, reflecting borderline appropriateness. Likewise, nearly all decision pathways in the CCE were deemed appropriate (median ≥7), except two: the pathway for Green DCR scores (indicating low likelihood of impairment) and a preliminary pathway for the anti-amyloid drug lecanemab, both of which garnered lower median ratings. Together, these findings suggest that the CCE’s guidance on cognitive impairment aligns well with expert clinical practice, whereas certain newer or non-cognitive recommendations require further refinement.

Inter-rater agreement was moderate at the per-patient level [median ICC(2,k) = 0.61; IQR 0.48–0.73], within the Koo and Li “moderate” band, and lower at the level of individual diagnostic-concern recommendations [median ICC(2,k) = 0.25]. The latter reflects both genuine specialty-level disagreement on borderline non-cognitive items—notably tremor, Parkinsonism, and cholinergic-pathway impairment—and reduced variance on items where the panel agreed near-unanimously, such as the LHQ high-risk recommendation. We caution that the per-patient ICC of 0.61, although moderate, is not strong, and that the within-rater pattern was influenced by a single expert who scored systematically lower than the others. ICC values in this range are expected when ratings include both true borderline clinical disagreement and restricted between-subject variance on items with near-unanimous endorsement (64, 65).

This pattern suggests that while experts might diverge on the necessity of individual recommendations—especially those with limited evidence or rare scenarios—they largely concur on how to proceed with an evaluation when viewing the patient holistically. The influence of a single outlier underscores a structural limitation: with only five experts, one expert’s opinions materially shift the metrics, and a larger panel would yield more robust consensus estimates. We also caution that variability among primary-care providers, the intended end-users of the CDS, will likely be greater than variability among the specialists rated here, which is a central motivation for the planned primary-care provider appropriateness study described under Next Steps below.

### Interpretability and clinician-facing transparency

Although the underlying DCR scoring uses ~2000 process features extracted from drawing and speech, the CDS recommendations presented to the clinician are produced by a rule-based engine that consumes interpretable, clinician-facing elements—the Green/Yellow/Red tier, the immediate and delayed-recall scores, the rendered command and copy clock images and their subscores, and the recall audio playback. The report does not surface the raw ~2000-feature vector; it surfaces the elements a clinician would normally examine on a paper Mini-Cog, augmented by automated, ML-enabled scoring. This design choice mirrors recommendations in the clinical decision support trust literature (38–41), which identifies transparent, auditable recommendation logic as central to adoption. The LHQ dementia-risk score is similarly transparent in its inputs: it integrates seven risk and protective factors, with weights calibrated against the LIBRA (56) and CAIDE (59) frameworks, and the high-risk recommendation maps to a prespecified threshold rather than an opaque cut point. Summaries of DCR feature families and LHQ inputs are provided in Supplementary Tables S1, S2, respectively.

### Cross-cultural acceptance of AI-assisted assessment

Although the present data were collected in the U.S. with English-speaking experts and patients, the global generalizability of AI-assisted digital cognitive assessment depends on cultural and individual factors that go beyond test psychometrics. Older adults’ acceptance of tablet-based and AI-enabled testing varies with prior digital exposure, education, language, and culturally specific attitudes toward technology and aging; some groups report anxiety, low self-efficacy, or skepticism toward AI-assisted health technologies (66–68). Mirroring this, healthcare professionals unfamiliar with AI-assisted tools may report low confidence or hesitation to act on automated recommendations (69–71). These attitudinal and competency factors will shape both the patient experience of tools such as the CCE and the uptake of CDS guidance in routine practice, and will need to be addressed through targeted education, multilingual support, and culturally adapted onboarding as the CCE is deployed in new settings.

Our results align with a growing body of literature emphasizing the value of digital cognitive assessment tools and CDS in primary care (14, 16, 26, 28, 72–77). Cognitive impairment remains underdetected in primary care—less than one-third of older adults report receiving routine cognitive screening, and over half of dementia cases are estimated to be missed or diagnosed late in general practice (77–79). Digital solutions like the CCE are designed to close this gap by enabling efficient screening coupled with actionable next steps. Since the present study was conducted, the CCE has received Class IIa certification under the European Union Medical Device Regulation (EU MDR) (80), which encompasses the CDS functionality of digital cognitive assessments such as the CCE (81).

Early evidence suggests primary care clinicians find such tools acceptable and useful. In a recent real-world usability study, primary care physicians (PCPs) implemented the CCE with minimal workflow disruption; 5/7 (71%) of providers reported that the assessment did not interfere with patient visits, and 6/7 (86%) indicated that the CCE’s results influenced their patient management. All participating PCPs in that study agreed with the CCE’s findings and expressed a willingness to continue using it (14). Similarly, a multi-clinic implementation project demonstrated the feasibility and impact of integrating digital cognitive screening at scale (82). Other digital cognitive assessment platforms have shown comparable benefits—for example, the TabCAT Brain Health Assessment was adopted by 95% of approached primary care clinicians and led to a significant increase in the detection of cognitive impairment (83). Our study adds a new dimension to this literature by demonstrating that the content of CDS recommendations in a machine-learning-enabled DCA can withstand specialist scrutiny.

The contrast in expert ratings between cognitive-focused and non-cognitive recommendations offers insight into where the CCE’s CDS is most effective and where caution is warranted. The greater variability in ratings for the Parkinsonism and tremor-related recommendations indicates that these recommendations should be interpreted with greater nuance. Some neurologists were unconvinced that subtle anomalies in a clock-drawing test alone provide sufficient evidence to justify an extensive workup for Parkinson’s disease or other movement disorders. This skepticism contrasts with published evidence linking digital clock-drawing performance to early Parkinsonian features (84–89) and may reflect limited familiarity among cognitive neurologists with the movement-disorder literature or a preference to corroborate motor findings before initiating a broader workup. In response to this input, we have revised those specific recommendations: the CDS now advises conducting a basic motor examination and considering referral to a neurologist only if the examination yields abnormal findings.

Similarly, the “cholinergic pathway impairment” concern received comparatively lower ratings. Although prior work has linked component placement patterns on the DCTclock to cholinergic system integrity (90), and reducing anticholinergic medication burden has been shown to improve clock-drawing performance (91), our thematic analysis confirmed that the supporting rationale was insufficiently transparent in the rating materials and may have been opaque to a generalist user. Accordingly, this recommendation should be accompanied by a clearer, clinically oriented rationale — specifically, how the observed cognitive pattern maps onto suspected basal forebrain cholinergic involvement (90) and refined wording that conveys the intended actionability. These refinements have been implemented in the production CDS.

### Anti-amyloid pathway

The preliminary anti-amyloid pathway (Figure 2), which received the lowest pathway-level rating (panel-pooled median 5), predated the publication of Appropriate Use Recommendations (AUR) for lecanemab and donanemab (5, 6). Expert comments clustered around four points: (i) the need for explicit blood-biomarker triage (e.g., plasma p-tau217 or Aβ42:40) before specialist referral; (ii) the importance of APOE-based eligibility filtering, particularly for ε4 homozygotes who carry the highest ARIA risk; (iii) the need for a baseline MRI for ARIA screening; and (iv) a defined ARIA monitoring schedule once therapy is initiated. The updated pathway, as implemented post-AUR, incorporates these elements and is depicted in Supplementary Figure S1 along with a summary of the changes. Re-evaluation of the updated pathway is planned as part of the next-stage validation program.

### Visuospatial memory, spatial navigation, and future digital cognitive assessment

The CCE’s current sensitivity is driven principally by memory (delayed recall) and executive/visuoconstructional (digital clock drawing) components, consistent with current evidence on the cognitive domains most affected in early Alzheimer’s disease. A growing literature, however, identifies visuospatial memory and spatial navigation as among the earliest-affected cognitive functions in preclinical and prodromal Alzheimer’s disease, reflecting medial temporal and entorhinal vulnerability (37). Incorporating brief, tablet-deliverable visuospatial and navigation tasks into the next generation of digital cognitive assessment—and into the CDS framework described here—is an active area of development and may further improve early detection in primary care.

Overall, the rating discrepancies highlight the areas where the CDS algorithm and messaging should be improved. The high ratings across cognitive-impairment recommendations reassure us that the core system is sound, while the mixed feedback on a few peripheral elements provides a roadmap for targeted enhancements.

### Limitations

Despite the encouraging results, this study has several limitations:

- *Expert panel size and composition*: Only five experts (all neurologists affiliated with academic centers) participated in the appropriateness ratings. Although a panel of 5–9 raters is consistent with RAND/UCLA Appropriateness Method conventions when raters are homogeneous in expertise (44), this small, specialized sample may not capture the full range of perspectives. The results are therefore sensitive to individual biases—indeed, one expert’s consistently lower ratings had a noticeable impact on certain scores. A larger panel would likely yield a more robust consensus and could mitigate the effect of any single outlier. Moreover, our experts were all cognitive or behavioral specialists, which limits the generalizability of our findings. They may be more familiar with dementia care guidelines than the average clinician, yet less familiar with movement disorders or other domains. Input from other specialties (e.g., geriatricians, psychiatrists, or movement disorder neurologists) and from frontline PCPs was not included, even though those providers would be using or interpreting the CDS in practice. The planned next-stage primary-care provider validation, described under Next Steps below, is designed to address this gap.
- Algorithm validation across speakers and drawers: The DCR’s automated delayed-recall scoring relies on an ASR pipeline. Although clinicians can verify and override the automated transcription via the report, performance in speakers with strong non-U.S. English accents and in patients with speech-motor disorders (e.g., dysarthria, post-stroke aphasia) has not been systematically evaluated, and broader multilingual and speech pathology validation is a planned next step. Drawing-based features have likewise been validated primarily in U.S. cohorts; the cross-cultural generalizability of clock-drawing kinematics remains an open question.
- *Generality of scenarios*: The study evaluated CDS recommendations using de-identified patient reports and fixed scenarios. Experts based their judgments on the information provided (CCE scores, risk factor questionnaire responses, and recommended actions) without having examined the patients themselves. In real clinical encounters, additional context—such as nuanced physical exam findings, longitudinal history, or patient preferences—could affect the perceived appropriateness of certain actions. Our findings indicate that the recommendations are appropriate in the idealized context of the CCE report, though this context may not fully capture real-world complexities.
- *Reliance on expert opinion vs. outcomes*: We assessed clinical appropriateness based on expert opinion rather than patient outcomes. While the RAND panel-style approach is a common method for validating clinical guidelines, it cannot determine whether adherence to CDS advice improves patient care. We do not yet know empirically whether implementing the CCE’s recommendations will lead to timelier interventions, improved patient/caregiver outcomes, or cost-effective care. Establishing the clinical impact of the CDS—in terms of reducing missed diagnoses or improving management—requires prospective outcome studies. Thus, our validation addresses one aspect (specialist agreement) but not ultimate patient benefit.
- *Potential biases*: Finally, the study was conducted by the CCE developers, and the experts were aware that they were evaluating a proprietary system. We took steps to mitigate bias (for example, experts had no financial stake in Linus Health and provided independent ratings), but an inherent risk remains that subtle expectations could influence findings. Additionally, the “preliminary” nature of certain pathways (e.g., the initial anti-amyloid therapy recommendation) means they were not fully developed, potentially contributing to lower ratings that may improve as the pathways are refined with the latest evidence.

These limitations suggest caution in interpreting and generalizing the results. The high appropriateness ratings are very encouraging, but they are an initial validation step. Further work is needed to confirm that the CCE’s recommendations are effective and applicable across diverse clinical settings.

### Next steps

This expert-review study is the first of a planned, multi-stage validation program for the CCE CDS. The next steps are: (i) a primary-care provider appropriateness study using the same RAND/UCLA framework with a larger and more diverse panel—general internists, family physicians, geriatricians, and advanced practice providers—recruited from both academic and community/safety-net settings, allowing direct comparison of specialist and primary-care judgements on identical reports; (ii) a prospective implementation study examining whether CDS recommendations change downstream clinical action (orders for laboratory workup, neuroimaging, referrals, and biomarker testing) and time-to-diagnosis for MCI and dementia; and (iii) an outcomes-anchored analysis using linked claims and electronic health record data to determine whether implementation of the CDS reduces the proportion of patients with delayed or missed MCI/dementia diagnoses. Where applicable, these studies will be pre-registered.

## Conclusion

In summary, this study provides initial evidence that the Linus Health CCE—a rapid, machine learning-enabled digital cognitive assessment coupled with clinical decision support—produces recommendations and diagnostic pathways that expert cognitive neurologists consider clinically appropriate. The high appropriateness ratings for all cognition-related recommendations and most pathways underscore the tool’s potential to meaningfully assist PCPs in the evaluation and management of cognitive impairment. By bridging the gap between a brief screening result and an actionable care plan, the CCE’s CDS functionality can enable PCPs to take well-founded next steps, even in settings where specialty expertise is not immediately available. Because inter-rater agreement among the specialist panel was moderate rather than strong, and because variability among primary-care end-users is likely to be greater, these findings should be interpreted as an initial, content-validity step rather than as definitive evidence of cross-population reliability. These findings support the clinical utility of integrating validated CDS algorithms into routine cognitive assessment workflows, ultimately helping to standardize and elevate the care for patients with or at risk for dementia. As healthcare systems grapple with rising dementia prevalence and as new disease-modifying treatments become available, tools that enable earlier detection and intervention will be increasingly vital. This validation is an encouraging first step toward that goal, reinforcing the significance and utility of the CCE’s recommendations for guiding clinical care for cognitive health, and motivating the planned next-stage primary-care provider validation.

## Data Availability

The raw data supporting the conclusions of this article will be made available by the authors, without undue reservation.
