# Supplementary material for: Initial specialist validation of clinical decision support recommendations from a machine learning-enabled digital cognitive assessment
Source: Front Neurol. 2026 Jun 17;17:1806000. doi: 10.3389/fneur.2026.1806000 (PMC13318572; doi:10.3389/fneur.2026.1806000)
Supplement: Supplementary file 1 [file Supplementary_file_1.docx]

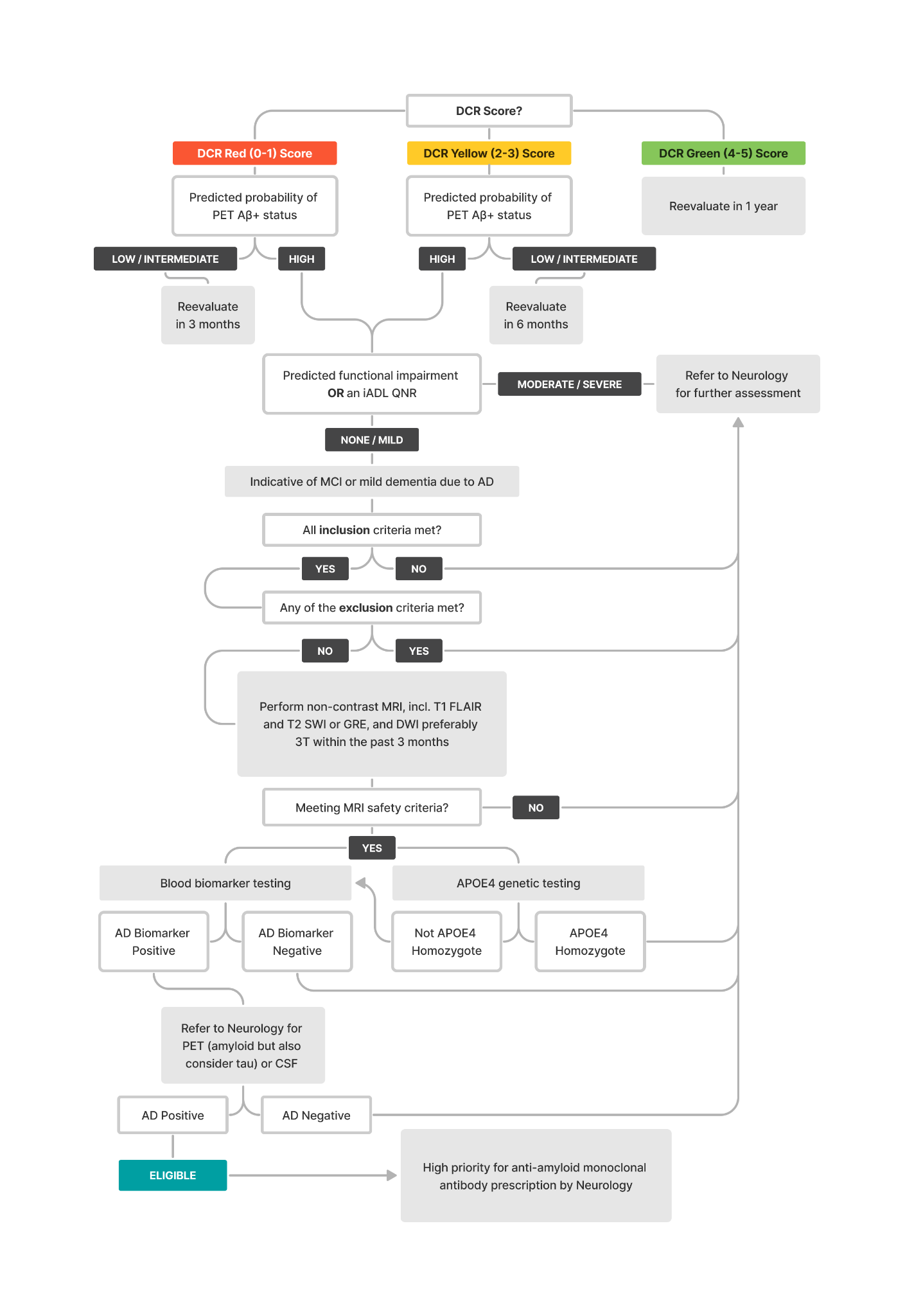


**Figure S1.** **Flowchart depicting the revised Clinical Decision Support (CDS) anti-amyloid therapy pathway, updated in response to the lecanemab and donanemab Appropriate Use Recommendations (AURs) published after the expert panel review was conducted.** Entry into the pathway is stratified by Digital Clock Drawing (DCR) risk score (Red [0–15], Yellow [2–30], or Green [4–15]), which predicts the probability of amyloid PET positivity; patients with low/intermediate predicted probability are flagged for reassessment in 3, 6, or 12 months, respectively, while those with high predicted probability continue through eligibility evaluation. The pathway incorporates four key updates aligned with current AURs: (1) **blood-biomarker triage** — plasma AD biomarker testing (e.g., p-tau217, Aβ42/40 ratio) is used as an initial screen to guide referral for confirmatory PET or CSF evaluation; (2) **refined APOE-based eligibility** — APOE ε4 genotyping is performed prior to treatment initiation, with APOE4 homozygosity flagged as a high-priority indicator for specialist neurology review given the substantially elevated risk of amyloid-related imaging abnormalities (ARIA); (3) **MRI exclusion criteria** — a non-contrast brain MRI (T1/FLAIR, T2*/GRE or SWI, and DWI; preferably 3T, obtained within the prior 3 months) is required to assess MRI safety criteria and screen for exclusionary findings (e.g., >4 cerebral microhemorrhages, cortical superficial siderosis, severe white matter disease, or imaging features consistent with CAA-related inflammation) before proceeding; and (4) **ARIA monitoring schedule** — consistent with published AURs, the pathway includes protocol-specified surveillance MRI timepoints to monitor for ARIA following treatment initiation. Patients meeting all eligibility criteria are referred to Neurology as candidates for anti-amyloid monoclonal antibody therapy. This updated pathway was implemented after the expert panel review reported in this study; the version evaluated during the review represented the preliminary iteration of the pathway prior to full AUR integration. AD, Alzheimer's disease; APOE, apolipoprotein E; ARIA, amyloid-related imaging abnormalities; CAA, cerebral amyloid angiopathy; CCE, Core Cognitive Evaluation; CDS, Clinical Decision Support; CSF, cerebrospinal fluid; DCR, Digital Clock Drawing; DWI, diffusion-weighted imaging; FLAIR, fluid-attenuated inversion recovery; GRE, gradient recalled echo; MCI, mild cognitive impairment; MRI, magnetic resonance imaging; PET, positron emission tomography; QNE, questionnaire of neurological examination; SWI, susceptibility-weighted imaging. Pathway criteria are informed by the lecanemab AUR (Cummings et al., 2023) and donanemab AUR (Rabinovici et al., 2025).

**References:**

Cummings J, Apostolova L, Rabinovici GD, Atri A, Aisen P, Greenberg S, Hendrix S, Selkoe D, Weiner M, Petersen RC, et al. Lecanemab: Appropriate Use Recommendations. J Prev Alz Dis (2023) doi: 10.14283/jpad.2023.30

Rabinovici GD, Selkoe DJ, Schindler SE, Aisen P, Apostolova LG, Atri A, Greenberg SM, Hendrix SB, Petersen RC, Weiner M, et al. Donanemab: Appropriate use recommendations. The Journal of Prevention of Alzheimer’s Disease (2025) 12:100150. doi: 10.1016/j.tjpad.2025.100150
