# Supplementary material for: Initial specialist validation of clinical decision support recommendations from a machine learning-enabled digital cognitive assessment
Source: Front Neurol. 2026 Jun 17;17:1806000. doi: 10.3389/fneur.2026.1806000 (PMC13318572; doi:10.3389/fneur.2026.1806000)
Supplement: Supplementary file 2 [file Table_1.docx]

| **Feature Family** | **Captured Signal / Modality** | **Cognitive Construct(s) Probed** | **Example Features** | **References** |
| --- | --- | --- | --- | --- |
| **Demographics** | Participant metadata | Age- and risk-related contextual variables that condition the prior probability of cognitive impairment and amyloid pathology | • Patient age (years)  • Sex / years of education (candidate covariates) | Jannati et al., 2025; Banks et al., 2024 |
| **Verbal recall accuracy** | Microphone audio → automatic speech recognition → rule-based scoring against target words | Verbal episodic memory; encoding, storage, and retrieval of recently presented information | • Delayed Recall score (0–3 words correctly recalled after a delay)  • Immediate Recall correct-words count (encoding check) | Jannati et al., 2025; Banks et al., 2024 |
| **Drawing kinematics (DCTclock — motor)** | Time-stamped (x, y) pen-stroke coordinates from the iPad/Apple Pencil during Command and Copy Clock | Simple and complex motor function; graphomotor control, pen-speed regulation, tremor/oscillation, and motor execution efficiency | • Average and maximum pen speed during clock face drawing  • Initiation and termination pen speed  • Oscillatory motion of the pen (deviation from a smooth trajectory)  • Percent ink time / total ink length / drawing size | Souillard-Mandar et al., 2021; Banks et al., 2024 |
| **Apple Pencil process metrics (DCTclock — motor)** | Stylus-sensor signals (e.g., altitude, azimuth, pressure) and derived temporal/spatial motor-activity envelopes. | Fine-grained motor planning, motor execution timing, and stroke-distribution regularity beyond what the 2-D pen trace alone captures. | • Apple Pencil skewness of stroke distances during DCTclock Copy Clock (asymmetry of stroke-length distribution)  • Apple Pencil time to reach 80% of horizontal motor activity during DCTclock Command Clock (how quickly the bulk of side-to-side drawing motion is completed) | Jannati et al., 2025 (and Supplement) |
| **Stroke-level timing / Information processing (DCTclock)** | Inter-stroke latencies, pauses, and pen-up / pen-down intervals derived from the time-stamped drawing. | Non-motor cognitive function; information-processing speed, planning, decision-making, attention, and executive control during drawing. | • Average inter-stroke latency; latency variability (log standard deviation)  • Longest single latency; count of notably long latencies  • Percent think time (time spent with pen off the page but task ongoing)  • COM/COP Information Processing composite scale | Souillard-Mandar et al., 2021; Banks et al., 2024 |
| **Spatial & symmetry metrics (DCTclock)** | Geometric analysis of the final drawn image after stroke-to-symbol classification (clock face, digits, hands). | Visuospatial reasoning and visuoconstructional skill; spatial planning, neglect/drift, geometric layout, and symbolic-element placement. | • DCTclock Command Clock component placement score (spatial accuracy of numbers and hands placed from instruction)  • DCTclock Copy Clock component placement score (spatial accuracy when copying)  • Clock-face circularity; drawing size; vertical and horizontal spatial placement on the page  • COM Spatial Reasoning composite scale | Souillard-Mandar et al., 2021; Banks et al., 2024 |
| **Speech timing (language)** | Recall audio segmented into speech vs. silence; ASR-derived word/syllable rates and pause statistics. | Lexical retrieval fluency, word-finding effort, and information-processing speed reflected in spoken output. | • Delayed Recall speech rate in words per second (how quickly the patient speaks while recalling)  • Long silent speech-pause count (≥ 2 s pauses during recall)  • Speech initiation time; short-pause count; speech rate per syllable | Banks et al., 2024; Jannati et al., 2025 |
| **Acoustic voice features** | Spectral and time-frequency analysis of recall audio waveform (e.g., librosa-derived features). | Voice quality and motor-speech function; subtle dysprosody, vocal-fold stability, and signal clarity associated with cognitive and neurodegenerative change. | • Delayed Recall median Cepstral Peak Prominence (CPP; voice-quality / signal-clarity measure)  • Fundamental frequency (F0) mean and variability  • Jitter (e.g., JitterPPQ5, JitterAbsolute) and shimmer  • Loudness statistics (mean, min, max, SD) | Banks et al., 2024; Jannati et al., 2025 |

**Table S1. Families of features extracted by the Digital Clock and Recall (DCR), with representative example features.** Composite scales (e.g., Drawing Efficiency, Information Processing, Simple/Complex Motor, Spatial Reasoning) are computed separately for the Command and Copy Clock conditions (Souillard-Mandar et al., 2021). ASR, automatic speech recognition; COM, Command Clock; COP, Copy Clock; CPP, Cepstral Peak Prominence; DCR, Digital Clock and Recall; DCTclock, Digital Clock Drawing Test; F0, fundamental frequency.

**References**

Banks R, Higgins C, Greene BR, Jannati A, Gomes-Osman J, Tobyne S, Bates D, Pascual-Leone A. Clinical classification of memory and cognitive impairment with multimodal digital biomarkers. Alzheimers Dement (Amst) (2024) 16:e12557. doi: 10.1002/dad2.12557

Jannati A, Thompson K, Toro-Serey C, Gomes-Osman J, Banks RE, Higgins C, Showalter J, Bates D, Tobyne S, Pascual-Leone A. Concurrent detection of cognitive impairment and amyloid positivity with a multimodal machine learning-enabled digital cognitive assessment. Alz Res Therapy (2025) 17:261. doi: 10.1186/s13195-025-01913-5

Souillard-Mandar W, Penney D, Schaible B, Pascual-Leone A, Au R, Davis R. DCTclock: Clinically-Interpretable and Automated Artificial Intelligence Analysis of Drawing Behavior for Capturing Cognition. Front. Digit. Health. 2021;3:750661. doi: 10.3389/fdgth.2021.750661
