# Supplementary material for: Initial specialist validation of clinical decision support recommendations from a machine learning-enabled digital cognitive assessment
Source: Front Neurol. 2026 Jun 17;17:1806000. doi: 10.3389/fneur.2026.1806000 (PMC13318572; doi:10.3389/fneur.2026.1806000)
Supplement: Supplementary file 3 [file Table_2.docx]

| **Domain / CCE Factor** | **Direction** | **Representative LHQ Inputs** | **Categorical Weight** | **Source / Reference(s)** |
| --- | --- | --- | --- | --- |
| **Panel A. Factors integrated into the CCE Dementia Risk Estimation (DRE) algorithm.** | | | | |
| **Cognitive activity**  *Modifiable; protective* | Protective | • “I do tasks that challenge my brain (like reading, writing, drawing, or playing a musical instrument) every day or almost every day.” (Yes / No) | **Large** | LIBRA (Schiepers et al., 2018); Ngandu et al., 2015; Neuffer et al., 2024; BBHI (Cattaneo et al., 2020). |
| **Depression**  *Modifiable; risk* | Risk | • “In the last month, I have been feeling… Down, depressed, hopeless / Little interest or pleasure in doing things / More tired or worn than usual.” (depression sub-items of the mood checklist) | **Large** | LIBRA (Schiepers et al., 2018); Lancet Commission (Livingston et al., 2020, 2024); Nedelec et al., 2022. |
| **Physical activity**  *Modifiable; mixed (active = protective; sedentary / frailty = risk)* | Mixed | • “I can walk 1 flight of stairs and/or walk 1 block without help.” (proxy for frailty)  • “On average, I sit more than six hours a day.” (sedentary behavior)  • “Over the past year, I have been doing some form of moderate or vigorous exercise at least three times a week.” (regular exercise) | **Intermediate** | LIBRA (Schiepers et al., 2018); CAIDE (Kivipelto et al., 2006); Lancet Commission (Livingston et al., 2020, 2024); FINGER (Ngandu et al., 2015); Ward et al., 2021. |
| **Diet**  *Modifiable; mixed* | Mixed | • “Please select the foods/drinks you have every day or on most days: Fruit / Vegetables / Red Meat / Alcohol / None.” (fruit and vegetable items contribute as protective; red-meat item as risk) | **Intermediate** | BBHI (Cattaneo et al., 2020); LIBRA (Schiepers et al., 2018); FINGER (Ngandu et al., 2015); Lancet Commission (Livingston et al., 2020, 2024); Neuffer et al., 2024. |
| **Physical health**  *Modifiable; risk (clinical comorbidities)* | Risk | • “A medical doctor has diagnosed me with: Diabetes / Hypertension / A heart rhythm problem / Vitamin deficiency (D or B12) / None.”  • “A medical doctor has told me that I should lose weight to improve my health.” (obesity proxy)  • “Over the last few years, I have lost more than 10 pounds unintentionally.” (unintentional weight loss / frailty) | **Intermediate** | LIBRA (Schiepers et al., 2018); CAIDE (Kivipelto et al., 2006); Lancet Commission (Livingston et al., 2020, 2024); FINGER (Ngandu et al., 2015); Stephen et al., 2019; Hendriks et al., 2023; Zhang et al., 2023; Zuliani et al., 2024. |
| **Smoking**  *Modifiable; risk* | Risk | • “I smoke cigarettes or vape regularly.” (Yes / No) | **Intermediate** | LIBRA (Schiepers et al., 2018); Lancet Commission (Livingston et al., 2020, 2024); Neuffer et al., 2024. |
| **Alcohol intake**  *Modifiable; risk at high intake* | Risk | • “Please select the foods/drinks you have every day or on most days: … Alcohol …” (daily-or-near-daily alcohol consumption item) | **Small** | LIBRA (Schiepers et al., 2018); Lancet Commission (Livingston et al., 2020, 2024); Neuffer et al., 2024. |
| **Panel B. Additional LHQ inputs that inform clinician-facing CDS recommendations and patient-facing brain-health education, but are not direct inputs to the DRE algorithm.** | | | | |
| **Subjective cognitive concern**  *Informs CDS recommendations* | Signal | • “I am concerned about changes in my memory or thinking abilities.” (Yes / No) | **—** | Abner et al., 2015; Rosalinde et al., 2019; Chan Lee et al., 2020; Wang et al., 2021. |
| **Sleep**  *Patient education + CDS* | Mixed | • “Please select all that apply about your sleep: I sleep on average between 7 and 8 hours each night / I typically take some time to fall asleep / I wake up during the night / None.” | **—** | BBHI (Cattaneo et al., 2020); Xu et al., 2020; Wong et al., 2023. |
| **Social isolation / engagement**  *Patient education + CDS* | Mixed | • “I sometimes feel lonely or that I lack company and support.”  • “Aside from my work, I am involved in some kind of association, club, choir, or volunteer activity at least once a week.” | **—** | Lancet Commission (Livingston et al., 2020, 2024); U.S. POINTER; BBHI (Cattaneo et al., 2020). |
| **Life meaning / purpose**  *Patient education* | Protective | • “I am generally satisfied with the course my life has taken.”  • “I know what I want to achieve in life, and what is my purpose.” | **—** | BBHI (Cattaneo et al., 2020); Bartrés-Faz et al., 2018; Sutin et al., 2023. |
| **Sensory loss**  *Hearing, vision, smell — CDS* | Risk | • “I have poor hearing or hearing problems.”  • “I have lost vision or developed vision problems.”  • “My sense of smell has gotten worse over the last 2–3 years.” | **—** | Lancet Commission (Livingston et al., 2020, 2024); Nedelec et al., 2022; Lad et al., 2022. |
| **Traumatic brain injury**  *CDS* | Risk | • “I have had a serious head injury (e.g., with loss of consciousness, hospitalization, or imaging indicating damage to the brain).” | **—** | Lancet Commission (Livingston et al., 2020, 2024). |
| **Air pollution**  *Patient education* | Risk | • “I live in an area of high levels of air pollution.” (Yes / No) | **—** | Lancet Commission (Livingston et al., 2020, 2024); Shi et al., 2023. |
| **Anxiety / Stress**  *CDS* | Risk | • “In the last month, I have been feeling: Anxious, nervous, worried / Stressed.” (anxiety and stress sub-items) | **—** | BBHI (Cattaneo et al., 2020); Wilson et al., 2007, 2011; Johansson et al., 2010. |
| **Medication adherence**  *CDS* | Signal | • “I am taking all my medications as prescribed.” (Yes / No / I do not take prescription medication) | **—** | LHQ design item (informs CDS recommendations regarding adherence). |

**Table S2. Life and Health Questionnaire (LHQ) Inputs and Weighting Overview.** Mapping of LHQ inputs to the seven risk and protective factors integrated by the Linus Health Core Cognitive Evaluation (CCE) Dementia Risk Estimation (DRE) algorithm, together with additional LHQ inputs that inform clinical decision support (CDS) recommendations and patient-facing brain-health education. To preserve the proprietary nature of the algorithm, factor contributions are summarized categorically as Large, Intermediate, or Small (by absolute magnitude, regardless of polarity). The categorical bands reflect the relative weighting framework calibrated against the LIBRA and CAIDE frameworks. The DRE integrates the seven **Panel A** factors via a proprietary algorithm informed by, but not identical to, LIBRA and CAIDE weights; an individual is flagged as at elevated risk of developing dementia if their estimated risk falls within the top quartile of the modeled population distribution (derived from Monte Carlo simulation). **Panel B** inputs are captured by the LHQ and surfaced to clinicians via the CDS recommendations and to patients via the personalized brain-health education report; they are not direct inputs to the DRE risk score and are therefore reported without a categorical weight (“—”). BBHI, Barcelona Brain Health Initiative; CAIDE, Cardiovascular Risk Factors, Aging, and Incidence of Dementia; CCE, Core Cognitive Evaluation; CDS, clinical decision support; DRE, Dementia Risk Estimation; FINGER, Finnish Geriatric Intervention Study to Prevent Cognitive Impairment and Disability; LHQ, Life and Health Questionnaire; LIBRA, Lifestyle for Brain Health; U.S. POINTER, U.S. Study to Protect Brain Health Through Lifestyle Intervention to Reduce Risk.

**Key References:**

Bartrés-Faz D, Cattaneo G, Solana J, Tormos JM, Pascual-Leone A. Meaning in life: resilience beyond reserve. *Alz Res Therapy* (2018) 10:47. doi: [10.1186/s13195-018-0381-z](https://doi.org/10.1186/s13195-018-0381-z)

Cattaneo G, Bartrés-Faz D, Morris TP, Sánchez JS, Macià D, Tarrero C, Tormos JM, Pascual-Leone A. The Barcelona Brain Health Initiative: A Cohort Study to Define and Promote Determinants of Brain Health. *Frontiers in Aging Neuroscience* (2018) 10: doi: [10.3389/fnagi.2018.00321](https://doi.org/10.3389/fnagi.2018.00321)

Ciesla M, Jannati A, Gomes Osman J, Alvaro P-L. Dementia Risk Estimation in the Linus Health Core Cognitive Evaluation. <https://linushealth.com/dementia-risk-estimation-in-the-linus-health-core-cognitive-evaluation> [Accessed May 1, 2023]

Hendriks S, Ranson JM, Peetoom K, Lourida I, Tai XY, de Vugt M, Llewellyn DJ, Köhler S. Risk factors for young-onset dementia in the UK Biobank. *JAMA Neurology* (2024) 81:134–142.<https://jamanetwork.com/journals/jamaneurology/article-abstract/2813439>

Kivipelto M, Ngandu T, Laatikainen T, Winblad B, Soininen H, Tuomilehto J. Risk score for the prediction of dementia risk in 20 years among middle aged people: a longitudinal, population-based study. *The Lancet Neurology* (2006) 5:735–741. doi: [10.1016/S1474-4422(06)70537-3](https://doi.org/10.1016/S1474-4422(06)70537-3)

Kivipelto M, Mangialasche F, Ngandu T. Lifestyle interventions to prevent cognitive impairment, dementia and Alzheimer disease. *Nature Reviews Neurology* (2018) 14:653–657. doi: [10.1038/s41582-018-0070-3](https://doi.org/10.1038/s41582-018-0070-3)

Lad EM, Mukherjee D, Stinnett SS, Cousins SW, Potter GG, Burke JR, Farsiu S, Whitson HE. Evaluation of inner retinal layers as biomarkers in mild cognitive impairment to moderate Alzheimer’s disease. *PLoS One* (2018) 13:e0192646. doi: [10.1371/journal.pone.0192646](https://doi.org/10.1371/journal.pone.0192646)

Livingston G, Huntley J, Sommerlad A, Ames D, Ballard C, Banerjee S, Brayne C, Burns A, Cohen-Mansfield J, Cooper C, et al. Dementia prevention, intervention, and care: 2020 report of the Lancet Commission. *Lancet* (2020) 396:413–446. doi: [10.1016/S0140-6736(20)30367-6](https://doi.org/10.1016/S0140-6736(20)30367-6)

Livingston G, Huntley J, Liu KY, Costafreda SG, Selbæk G, Alladi S, Ames D, Banerjee S, Burns A, Brayne C, et al. Dementia prevention, intervention, and care: 2024 report of the Lancet standing Commission. *The Lancet* (2024) 0: doi: [10.1016/S0140-6736(24)01296-0](https://doi.org/10.1016/S0140-6736(24)01296-0)

Nedelec T, Couvy-Duchesne B, Monnet F, Daly T, Ansart M, Gantzer L, Lekens B, Epelbaum S, Dufouil C, Durrleman S. Identifying health conditions associated with Alzheimer’s disease up to 15 years before diagnosis: an agnostic study of French and British health records. *Lancet Digit Health* (2022) 4:e169–e178. doi: [10.1016/S2589-7500(21)00275-2](https://doi.org/10.1016/S2589-7500(21)00275-2)

Neuffer J, Wagner M, Moreno E, Le Grand Q, Mishra A, Trégouët D-A, Leffondre K, Proust-Lima C, Foubert-Samier A, Berr C, et al. Association of LIfestyle for BRAin health risk score (LIBRA) and genetic susceptibility with incident dementia and cognitive decline. *Alzheimer’s & Dementia* (2024) 20:4250–4259. doi: [10.1002/alz.13801](https://doi.org/10.1002/alz.13801)

Ngandu T, Lehtisalo J, Solomon A, Levälahti E, Ahtiluoto S, Antikainen R, Bäckman L, Hänninen T, Jula A, Laatikainen T, et al. A 2 year multidomain intervention of diet, exercise, cognitive training, and vascular risk monitoring versus control to prevent cognitive decline in at-risk elderly people (FINGER): a randomised controlled trial. *The Lancet* (2015) 385:2255–2263. doi: [10.1016/S0140-6736(15)60461-5](https://doi.org/10.1016/S0140-6736(15)60461-5)

Schiepers OJG, Köhler S, Deckers K, Irving K, O’Donnell CA, van den Akker M, Verhey FRJ, Vos SJB, de Vugt ME, van Boxtel MPJ. Lifestyle for Brain Health (LIBRA): a new model for dementia prevention. *Int J Geriatr Psychiatry* (2018) 33:167–175. doi: [10.1002/gps.4700](https://doi.org/10.1002/gps.4700)

Shi L, Zhu Q, Wang Y, Hao H, Zhang H, Schwartz J, Amini H, Van Donkelaar A, Martin RV, Steenland K, et al. Incident dementia and long-term exposure to constituents of fine particle air pollution: A national cohort study in the United States. *Proc Natl Acad Sci USA* (2023) 120:e2211282119. doi: [10.1073/pnas.2211282119](https://doi.org/10.1073/pnas.2211282119)

Stephen R, Liu Y, Ngandu T, Antikainen R, Hulkkonen J, Koikkalainen J, Kemppainen N, Lötjönen J, Levälahti E, Parkkola R, et al. Brain volumes and cortical thickness on MRI in the Finnish Geriatric Intervention Study to Prevent Cognitive Impairment and Disability (FINGER). *Alzheimers Res Ther* (2019) 11:53. doi: [10.1186/s13195-019-0506-z](https://doi.org/10.1186/s13195-019-0506-z)

Sutin AR, Stephan Y, Luchetti M, Terracciano A. Purpose in life and markers of immunity and inflammation: Testing pathways of episodic memory. *J Psychosom Res* (2023) 174:111487. doi: [10.1016/j.jpsychores.2023.111487](https://doi.org/10.1016/j.jpsychores.2023.111487)

Ward DD, Wallace LMK, Rockwood K. Frailty and Risk of Dementia in Mild Cognitive Impairment Subtypes. *Annals of Neurology* (2021) 89:1221–1225. doi: [10.1002/ana.26064](https://doi.org/10.1002/ana.26064)

Wong R, Lovier MA. Sleep Disturbances and Dementia Risk in Older Adults: Findings From 10 Years of National U.S. Prospective Data. *American Journal of Preventive Medicine* (2023) 64:781–787. doi: [10.1016/j.amepre.2023.01.008](https://doi.org/10.1016/j.amepre.2023.01.008)

Xu W, Tan C-C, Zou J-J, Cao X-P, Tan L. Sleep problems and risk of all-cause cognitive decline or dementia: an updated systematic review and meta-analysis. *J Neurol Neurosurg Psychiatry* (2020) 91:236–244. doi: [10.1136/jnnp-2019-321896](https://doi.org/10.1136/jnnp-2019-321896)

Zhang W, Liang J, Li C, Gao D, Ma Q, Pan Y, Wang Y, Xie W, Zheng F. Age at Diagnosis of Atrial Fibrillation and Incident Dementia. *JAMA Network Open* (2023) 6:e2342744. doi: [10.1001/jamanetworkopen.2023.42744](https://doi.org/10.1001/jamanetworkopen.2023.42744)

Zuliani G, Brombo G, Polastri M, Romagnoli T, Mola G, Riccetti R, Seripa D, Trentini A, Cervellati C. High plasma homocysteine levels predict the progression from mild cognitive impairment to dementia. *Neurochemistry International* (2024) 177:105763. doi: [10.1016/j.neuint.2024.105763](https://doi.org/10.1016/j.neuint.2024.105763)
