# Supplementary material for: Initial specialist validation of clinical decision support recommendations from a machine learning-enabled digital cognitive assessment
Source: Front Neurol. 2026 Jun 17;17:1806000. doi: 10.3389/fneur.2026.1806000 (PMC13318572; doi:10.3389/fneur.2026.1806000)
Supplement: Supplementary file 4 [file Table_3.docx]

| **Characteristic** | **Value (N = 5)** |
| --- | --- |
| **Panel composition** | |
| Experts invited and enrolled | 7 |
| Withdrawn (competing obligations) | 2 |
| Completed ratings within study timeline (analytic panel) | 5 (100%) |
| **Training and board certification** | |
| Medical degree (MD) | 5 / 5 (100%) |
| U.S. ACGME-accredited Neurology residency | 5 / 5 (100%) |
| ABPN board certification in Neurology | 5 / 5 (100%) |
| Fellowship training in cognitive/behavioral neurology, behavioral neurology and neuropsychiatry (BNNP), or aging and dementia | 5 / 5 (100%) |
| UCNS subspecialty certification in Behavioral Neurology & Neuropsychiatry | 3 / 5 (60%) |
| UCNS subspecialty certification in Geriatric Neurology | 1 / 5 (20%) |
| Additional graduate degree (MA, MS, or PhD) | 3 / 5 (60%) |
| **Academic affiliation and rank** | |
| Primary affiliation with a U.S. academic medical center | 5 / 5 (100%) |
| Institutions represented | Harvard Medical School (BIDMC, BWH)  Perelman School of Medicine, University of Pennsylvania  Vagelos College of Physicians and Surgeons, Columbia University |
| Academic rank at primary U.S. institution | Full Professor of Neurology: 2  Associate Professor of Neurology: 1  Assistant Professor of Neurology: 2 |
| **Clinical experience** | |
| Active outpatient or consultative practice focused on cognitive disorders during the rating period | 5 / 5 (100%) |
| Years of post-fellowship clinical experience (range) | 4 – 49 years (median ≈ 19) |
| Faculty appointment in a memory disorders, behavioral neurology, brain-mind, or Alzheimer's disease center/program | 5 / 5 (100%) |
| Documented clinical activity in safety-net, rural, free-clinic, veterans (VA), or otherwise underserved populations (current or earlier-career) | 5 / 5 (100%) |
| **Geographic distribution (U.S. practice site)** | |
| U.S. region | Northeast: 5 / 5 (100%) |
| U.S. states represented | MA (2), PA (2), NY (1) |
| **Scholarly contributions and external recognition** | |
| Peer-reviewed publications in cognitive/behavioral neurology, dementia, or Alzheimer's disease | 5 / 5 (100%) |
| History of extramurally funded research as PI or Co-I (e.g., NIH/NIA, foundation, industry-sponsored clinical trials, or equivalent international funder) | 4 / 5 (80%) |
| Authorship of book chapters or textbooks on cognitive/behavioral neurology (e.g., Merritt's Textbook of Neurology, Samuels's Manual of Neurologic Therapeutics, Geriatric Neurology, Principles of Frontal Lobe Function) | 4 / 5 (80%) |
| Contribution to clinical practice guidelines, consensus statements, or expert position papers (e.g., AAN Guidelines Subcommittee, AAN Behavioral Neurology Section workgroup, EADC consensus, Alzheimer Europe Expert Advisory Panel, IOM/NASEM review, UpToDate authorship) | 4 / 5 (80%) |
| Editorial role at a peer-reviewed journal (editor-in-chief, senior, associate, section, or editorial-board member) | 4 / 5 (80%) |
| **Professional society engagement** | |
| Member, American Academy of Neurology (AAN) | 5 / 5 (100%) |
| Fellow of the AAN (FAAN) | 2 / 5 (40%) |
| Fellow of the American Neurological Association (FANA) | 1 / 5 (20%) |
| Documented leadership role in a national or international professional society, consortium, or specialty section (e.g., Society for Behavioral and Cognitive Neurology Executive Committee or Presidency; AAN BNNP Section Chair; EADC Executive Committee; UCNS Subspecialty Examination Committee; Academy of Aphasia Scientific Meeting Committee Chair) | 4 / 5 (80%) |
| Recipient of a named lectureship, society award, or honor relevant to behavioral/cognitive neurology or stroke (e.g., AAN Norman Geschwind Prize, AAN Changemaker Award, American Stroke Association C. Miller Fisher Award, Arnold P. Gold Foundation Award) | 3 / 5 (60%) |
| **Conflict of interest** | |
| Current or prior affiliation, collaboration, or financial interest in Linus Health | 0 / 5 (0%) |
| Compensation for participation in this study | Honoraria for time only |

**Table S3. Aggregate characteristics of the Clinical Expert Panel (N = 5).** All panelists were independently recruited under predefined eligibility criteria, including U.S. board certification in neurology with specialty practice in cognitive/behavioral neurology, primary affiliation with an academic medical center, an active evidence-based clinical practice focused on cognitive disorders, and no current or prior affiliation, collaboration, or financial interest in Linus Health. Values are presented in aggregate to preserve panelist anonymity, consistent with RAND/UCLA Appropriateness Method reporting conventions. Note: All values were extracted from CVs provided by each panelist. Geographic distribution reflects the U.S. practice site of each panelist at the time of the rating exercise. "Underserved practice" is operationalized here as documented clinical activity, programmatic leadership, or volunteer service addressing safety-net, rural, free-clinic, veterans (VA), care-access, or minority-aging populations during the panelist's career. AAN, American Academy of Neurology; ABPN, American Board of Psychiatry and Neurology; ACGME, Accreditation Council for Graduate Medical Education; ACTC, Alzheimer Clinical Trial Consortium; BIDMC, Beth Israel Deaconess Medical Center; BNNP, Behavioral Neurology and Neuropsychiatry; BWH, Brigham and Women's Hospital; CEP, Clinical Expert Panel; Co-I, co-investigator; EADC, European Alzheimer's Disease Consortium; FAAN, Fellow of the American Academy of Neurology; FANA, Fellow of the American Neurological Association; IOM, Institute of Medicine; NASEM, National Academies of Sciences, Engineering, and Medicine; NIA, National Institute on Aging; NIH, National Institutes of Health; PI, principal investigator; UCNS, United Council for Neurologic Subspecialties; VA, Veterans Affairs.
