# Supplementary material for: Initial specialist validation of clinical decision support recommendations from a machine learning-enabled digital cognitive assessment
Source: Front Neurol. 2026 Jun 17;17:1806000. doi: 10.3389/fneur.2026.1806000 (PMC13318572; doi:10.3389/fneur.2026.1806000)
Supplement: Supplementary file 5 [file Table_4.docx]

| **Reviewer** | **Median** | **SD** | **Lower Quartile** | **Upper Quartile** | **IQR** |
| --- | --- | --- | --- | --- | --- |
| 1 | 6.5 | 2.03 | 4 | 8.00 | 4.00 |
| 2 | 6.5 | 2.32 | 5 | 9.00 | 4.00 |
| 3 | 4.5 | 2.33 | 3 | 7.75 | 4.75 |
| 4 | 9.0 | 1.10 | 7 | 9.00 | 2.00 |
| 5 | 8.0 | 1.34 | 7 | 9.00 | 2.00 |

**Table S4.** Concerns’ median ratings per rater.
