# Supplementary material for: Initial specialist validation of clinical decision support recommendations from a machine learning-enabled digital cognitive assessment
Source: Front Neurol. 2026 Jun 17;17:1806000. doi: 10.3389/fneur.2026.1806000 (PMC13318572; doi:10.3389/fneur.2026.1806000)
Supplement: Supplementary file 6 [file Table_5.docx]

| **Concern** | **Median** | **SD** | **Lower Quantile** | **Upper quantile** | **IQR** |
| --- | --- | --- | --- | --- | --- |
| Cholinergic Pathway Impairment | 5.5 | 2.38 | 4.25 | 8.0 | 3.75 |
| Concern for Tremor | 5.0 | 2.04 | 3.00 | 7.0 | 4.00 |
| Executive Mixed Domain Impairment | 9.0 | 1.77 | 8.00 | 9.0 | 1.00 |
| Executive Vascular Cognitive Impairment | 7.0 | 1.69 | 6.50 | 8.0 | 1.50 |
| LHQ High Risk | 9.0 | 1.00 | 8.00 | 9.0 | 1.00 |
| Mixed Domain Impairment | 8.0 | 1.52 | 7.00 | 9.0 | 2.00 |
| No Concerns | 9.0 | 0.70 | 8.25 | 9.0 | 0.75 |
| Parkinsonism | 5.0 | 1.93 | 3.00 | 7.0 | 4.00 |
| Verbal Memory Impairment | 8.0 | 0.56 | 8.00 | 8.5 | 0.50 |
| Verbal Memory Mixed Domain Impairment | 7.0 | 1.97 | 6.00 | 8.0 | 2.00 |

**Table S5.** Concerns’ median ratings per concern
