# Supplementary material for: Initial specialist validation of clinical decision support recommendations from a machine learning-enabled digital cognitive assessment
Source: Front Neurol. 2026 Jun 17;17:1806000. doi: 10.3389/fneur.2026.1806000 (PMC13318572; doi:10.3389/fneur.2026.1806000)
Supplement: Supplementary file 7 [file Table_6.docx]

| **Patient** | **Median** | **SD** | **Lower Quartile** | **Upper Quartile** | **IQR** |
| --- | --- | --- | --- | --- | --- |
| 1 | 6.0 | 1.64 | 5.00 | 7.25 | 2.25 |
| 2 | 7.0 | 2.07 | 5.00 | 8.00 | 3.00 |
| 3 | 7.0 | 2.37 | 5.00 | 8.75 | 3.75 |
| 4 | 6.5 | 2.08 | 5.00 | 8.00 | 3.00 |
| 5 | 8.0 | 1.96 | 8.00 | 8.75 | 0.75 |
| 6 | 7.0 | 2.46 | 3.50 | 8.00 | 4.50 |
| 7 | 6.0 | 1.91 | 5.00 | 8.00 | 3.00 |
| 8 | 9.0 | 0.89 | 8.00 | 9.00 | 1.00 |
| 9 | 7.0 | 2.07 | 7.00 | 8.50 | 1.50 |
| 10 | 7.0 | 2.16 | 5.25 | 8.75 | 3.50 |
| 11 | 8.0 | 2.26 | 5.00 | 9.00 | 4.00 |
| 12 | 6.0 | 2.26 | 4.25 | 7.75 | 3.50 |
| 13 | 7.0 | 2.11 | 5.00 | 8.50 | 3.50 |
| 14 | 8.0 | 2.50 | 4.50 | 8.50 | 4.00 |
| 15 | 7.0 | 2.56 | 3.75 | 8.25 | 4.50 |
| 16 | 9.0 | 1.34 | 8.25 | 9.00 | 0.75 |
| 17 | 8.5 | 1.99 | 6.50 | 9.00 | 2.50 |
| 18 | 8.0 | 1.80 | 6.00 | 9.00 | 3.00 |
| 19 | 7.0 | 2.61 | 4.00 | 8.50 | 4.50 |
| 20 | 4.0 | 2.22 | 3.00 | 6.75 | 3.75 |
| 21 | 9.0 | 0.45 | 9.00 | 9.00 | 0.00 |

**Table S6. Concerns’ median ratings per putative patient.**
