# Supplementary material for: Initial specialist validation of clinical decision support recommendations from a machine learning-enabled digital cognitive assessment
Source: Front Neurol. 2026 Jun 17;17:1806000. doi: 10.3389/fneur.2026.1806000 (PMC13318572; doi:10.3389/fneur.2026.1806000)
Supplement: Supplementary file 8 [file Table_7.docx]

| **Reviewer** | **Median** | **SD** | **Lower Quartile** | **Upper Quartile** | **IQR** |
| --- | --- | --- | --- | --- | --- |
| 1 | 8 | 1.30 | 6 | 8 | 2 |
| 2 | 7 | 0.33 | 7 | 7 | 0 |
| 3 | 7 | 1.90 | 5 | 8 | 3 |
| 4 | 7 | 0.97 | 6 | 7 | 1 |
| 5 | 7 | 0.88 | 7 | 7 | 0 |

**Table S7.** Pathway summary statistics per rater.
