# Supplementary material for: Initial specialist validation of clinical decision support recommendations from a machine learning-enabled digital cognitive assessment
Source: Front Neurol. 2026 Jun 17;17:1806000. doi: 10.3389/fneur.2026.1806000 (PMC13318572; doi:10.3389/fneur.2026.1806000)
Supplement: Supplementary file 9 [file Table_8.docx]

| **Pathway** | **Median** | **SD** | **Lower Quartile** | **Upper Quartile** | **IQR** |
| --- | --- | --- | --- | --- | --- |
| Overall | 7 | 0.89 | 7 | 7 | 0 |
| Green | 6 | 0.84 | 6 | 7 | 1 |
| Yellow | 7 | 1.52 | 7 | 7 | 0 |
| Yellow HCRC | 7 | 0.84 | 6 | 7 | 1 |
| Yellow IR3RCDB | 7 | 2.07 | 7 | 8 | 1 |
| Red | 7 | 1.10 | 7 | 7 | 0 |
| Red IRLOWER3HC | 7 | 0.84 | 7 | 8 | 1 |
| Red Hearing | 8 | 0.84 | 7 | 8 | 1 |
| Lecanemab | 5 | 1.10 | 5 | 7 | 2 |

**Table S8. Summary statistics per pathway.** Pathway labels are abbreviated as follows: Red IR3DB (Immediate recall of 3 with downstream branches) refers to the pathway triggered by a red DCR score (0–1) and an Immediate Recall score equal to 3; Red IRLOWER3HC (Immediate recall lower than 3, with hearing concern) refers to the pathway triggered by a red DCR score and an Immediate Recall score lower than 3 (with hearing concerns and reversible-causes nodes); Yellow IR3RCDB (Immediate recall 3, reversible causes and downstream branches) refers to the pathway triggered by a yellow DCR score (2–3) and an Immediate Recall score equal to 3 (with reversible-causes and downstream branches); Yellow HCRC (Hearing concerns and reversible with reversible causes) refers to the pathway triggered by a yellow DCR score with an Immediate Recall score less than 3 (with hearing-concerns and reversible-causes nodes).
