# Supplementary material for: Initial specialist validation of clinical decision support recommendations from a machine learning-enabled digital cognitive assessment
Source: Front Neurol. 2026 Jun 17;17:1806000. doi: 10.3389/fneur.2026.1806000 (PMC13318572; doi:10.3389/fneur.2026.1806000)
Supplement: Supplementary file 10 [file Table_9.docx]

| **Concern** | **N** | **ICC Type** | **ICC Score** | **F** | **df1** | **df2** | **p-value** | **95% CI (lower)** | **95% CI (upper)** |
| --- | --- | --- | --- | --- | --- | --- | --- | --- | --- |
| Verbal Memory Mixed Domain Impairment | 5 | ICC2 | 0.20 | 3.99 | 4 | 16 | 0.019 | 0.00 | 0.73 |
|  | 5 | ICC2k | 0.55 | 3.99 | 4 | 16 | 0.019 | 0.00 | 0.93 |
| Executive Mixed Domain Impairment | 3 | ICC2 | 0.02 | 1.15 | 2 | 8 | 0.364 | -0.13 | 0.86 |
|  | 3 | ICC2k | 0.09 | 1.15 | 2 | 8 | 0.364 | -1.27 | 0.97 |
| Verbal Memory Impairment | 3 | ICC2 | 0.20 | 2.25 | 2 | 8 | 0.167 | -0.13 | 0.95 |
|  | 3 | ICC2k | 0.56 | 2.25 | 2 | 8 | 0.167 | -1.34 | 0.99 |
| Mixed Domain Impairment | 4 | ICC2 | 0.23 | 2.56 | 3 | 12 | 0.103 | -0.08 | 0.87 |
|  | 4 | ICC2k | 0.60 | 2.56 | 3 | 12 | 0.103 | -0.57 | 0.97 |
| Executive Vascular Cognitive Impairment | 3 | ICC2 | 0.09 | 2.20 | 2 | 8 | 0.173 | -0.05 | 0.87 |
|  | 3 | ICC2k | 0.33 | 2.20 | 2 | 8 | 0.1735 | -0.30 | 0.97 |
| LHQ32 High Risk | 8 | ICC2 | 0.00 | 1.00 | 7 | 28 | 0.451 | -0.10 | 0.32 |
|  | 8 | ICC2k | 0.00 | 1.00 | 7 | 28 | 0.451 | -0.81 | 0.70 |
| Cholinergic Pathway Impairment | 14 | ICC2 | 0.04 | 1.57 | 13 | 52 | 0.124 | -0.02 | 0.21 |
|  | 14 | ICC2k | 0.18 | 1.57 | 13 | 52 | 0.124 | -0.14 | 0.57 |
| Parkinsonism | 9 | ICC2 | 0.00 | 1.00 | 8 | 32 | 0.455 | -0.04 | 0.14 |
|  | 9 | ICC2k | 0.00 | 1.00 | 8 | 32 | 0.455 | -0.21 | 0.45 |
| Concern for Tremor | 7 | ICC2 | 0.01 | 1.12 | 6 | 24 | 0.378 | -0.04 | 0.24 |
|  | 7 | ICC2k | 0.04 | 1.12 | 6 | 24 | 0.378 | -0.25 | 0.61 |
| No concerns detected | 2 | ICC2 | 0.09 | 2.67 | 1 | 4 | 0.177 | -0.04 | 0.99 |
|  | 2 | ICC2k | 0.33 | 2.67 | 1 | 4 | 0.177 | -0.24 | 1.00 |

**Table S9.** ICCs (2 and 2k) per concern.
