# Supplementary material for: Initial specialist validation of clinical decision support recommendations from a machine learning-enabled digital cognitive assessment
Source: Front Neurol. 2026 Jun 17;17:1806000. doi: 10.3389/fneur.2026.1806000 (PMC13318572; doi:10.3389/fneur.2026.1806000)
Supplement: Supplementary file 11 [file Table_10.docx]

| **Patient** | **Concerns** | **ICC Type** | **ICC Score** | **F** | **df1** | **df2** | **p-value** | **95% CI (lower)** | **95% CI (upper)** |
| --- | --- | --- | --- | --- | --- | --- | --- | --- | --- |
| 1 | 4 | ICC2 | 0.12 | 1.81 | 3 | 12 | 0.199 | -0.10 | 0.80 |
|  | 4 | ICC2k | 0.40 | 1.81 | 3 | 12 | 0.199 | -0.87 | 0.95 |
| 2 | 3 | ICC2 | 0.24 | 11.43 | 2 | 8 | 0.0045 | 0.02 | 0.93 |
|  | 3 | ICC2k | 0.61 | 11.43 | 2 | 8 | 0.0045 | 0.07 | 0.99 |
| 3 | 2 | ICC2 | 0.00 | 1.00 | 1 | 4 | 0.3739 | -0.22 | 0.99 |
|  | 2 | ICC2k | 0.00 | 1.00 | 1 | 4 | 0.3739 | -11.22 | 1.00 |
| 4 | 4 | ICC2 | 0.36 | 5.57 | 3 | 12 | 0.0125 | 0.03 | 0.91 |
|  | 4 | ICC2k | 0.74 | 5.57 | 3 | 12 | 0.0125 | 0.15 | 0.98 |
| 5 | 2 | ICC2 | 0.09 | 1.56 | 1 | 4 | 0.2803 | -0.16 | 1.00 |
|  | 2 | ICC2k | 0.32 | 1.56 | 1 | 4 | 0.2803 | -2.36 | 1.00 |
| 6 | 3 | ICC2 | 0.56 | 13.45 | 2 | 8 | 0.0027 | 0.12 | 0.98 |
|  | 3 | ICC2k | 0.87 | 13.45 | 2 | 8 | 0.0027 | 0.41 | 1.00 |
| 7 | 3 | ICC2 | 0.54 | 11.36 | 2 | 8 | 0.0045 | 0.10 | 0.98 |
|  | 3 | ICC2k | 0.85 | 11.36 | 2 | 8 | 0.0045 | 0.37 | 1.00 |
| 9 | 3 | ICC2 | 0.42 | 8.93 | 2 | 8 | 0.0091 | 0.05 | 0.97 |
|  | 3 | ICC2k | 0.79 | 8.93 | 2 | 8 | 0.0091 | 0.20 | 0.99 |
| 10 | 2 | ICC2 | 0.21 | 3.45 | 1 | 4 | 0.1369 | -0.06 | 1.00 |
|  | 2 | ICC2k | 0.58 | 3.45 | 1 | 4 | 0.1369 | -0.43 | 1.00 |
| 11 | 4 | ICC2 | 0.37 | 6.43 | 3 | 12 | 0.0076 | 0.05 | 0.91 |
|  | 4 | ICC2k | 0.75 | 6.43 | 3 | 12 | 0.0076 | 0.19 | 0.98 |
| 12 | 2 | ICC2 | 0.15 | 3.37 | 1 | 4 | 0.1403 | -0.04 | 1.00 |
|  | 2 | ICC2k | 0.48 | 3.37 | 1 | 4 | 0.1403 | -0.27 | 1.00 |
| 13 | 3 | ICC2 | 0.44 | 7.73 | 2 | 8 | 0.0135 | 0.05 | 0.97 |
|  | 3 | ICC2k | 0.80 | 7.73 | 2 | 8 | 0.0135 | 0.20 | 0.99 |
| 14 | 3 | ICC2 | 0.24 | 3.16 | 2 | 8 | 0.0976 | -0.06 | 0.95 |
|  | 3 | ICC2k | 0.61 | 3.16 | 2 | 8 | 0.0976 | -0.39 | 0.99 |
| 15 | 4 | ICC2 | 0.33 | 6.48 | 3 | 12 | 0.0074 | 0.03 | 0.89 |
|  | 4 | ICC2k | 0.71 | 6.48 | 3 | 12 | 0.0074 | 0.14 | 0.98 |
| 16 | 2 | ICC2 | 0.11 | 1.67 | 1 | 4 | 0.2662 | -0.17 | 1.00 |
|  | 2 | ICC2k | 0.37 | 1.67 | 1 | 4 | 0.2662 | -2.49 | 1.00 |
| 17 | 4 | ICC2 | 0.34 | 5.12 | 3 | 12 | 0.0164 | 0.02 | 0.90 |
|  | 4 | ICC2k | 0.72 | 5.12 | 3 | 12 | 0.0164 | 0.11 | 0.98 |
| 18 | 3 | ICC2 | 0.13 | 2.25 | 2 | 8 | 0.1677 | -0.07 | 0.91 |
|  | 3 | ICC2k | 0.43 | 2.25 | 2 | 8 | 0.1677 | -0.51 | 0.98 |
| 19 | 3 | ICC2 | 0.26 | 4.17 | 2 | 8 | 0.0575 | -0.02 | 0.95 |
|  | 3 | ICC2k | 0.64 | 4.17 | 2 | 8 | 0.0575 | -0.13 | 0.99 |
| 20 | 2 | ICC2 | 0.00 | 1.00 | 1 | 4 | 0.3739 | -0.04 | 0.97 |
|  | 2 | ICC2k | 0.00 | 1.00 | 1 | 4 | 0.3739 | -0.20 | 0.99 |

**Table S10.** Concerns’ ICC scores (2k and 2,1) per patient. Patient 21 was excluded because it only contained two diagnostic concerns.
